# microCLIP super learning framework uncovers functional transcriptome-wide miRNA interactions

**DOI:** 10.1038/s41467-018-06046-y

**Published:** 2018-09-06

**Authors:** Maria D. Paraskevopoulou, Dimitra Karagkouni, Ioannis S. Vlachos, Spyros Tastsoglou, Artemis G. Hatzigeorgiou

**Affiliations:** 1 DIANA-Lab, Department of Electrical & Computer Engineering, University of Thessaly, 38221 Volos, Greece; 2 Molecular Oncology Research Institute, Tufts Medical Center, Boston, 02111 MA, USA; 3 Department of Neurology, Brigham & Women’s Hospital, Harvard Medical School, Boston, 02115 MA USA; 4 Hellenic Pasteur Institute, 127 Vasilissis Sofias Avenue, 11521 Athens, Greece

## Abstract

Argonaute crosslinking and immunoprecipitation (CLIP) experiments are the most widely used high-throughput methodologies for miRNA targetome characterization. The analysis of Photoactivatable Ribonucleoside-Enhanced (PAR) CLIP methodology focuses on sequence clusters containing T-to-C conversions. Here, we demonstrate for the first time that the non-T-to-C clusters, frequently observed in PAR-CLIP experiments, exhibit functional miRNA-binding events and strong RNA accessibility. This discovery is based on the analysis of an extensive compendium of bona fide miRNA-binding events, and is further supported by numerous miRNA perturbation experiments and structural sequencing data. The incorporation of these previously neglected clusters yields an average of 14% increase in miRNA-target interactions per PAR-CLIP library. Our findings are integrated in microCLIP (www.microrna.gr/microCLIP), a cutting-edge framework that combines deep learning classifiers under a super learning scheme. The increased performance of microCLIP in CLIP-Seq-guided detection of miRNA interactions, uncovers previously elusive regulatory events and miRNA-controlled pathways.

## Abstract (web summary)

AGO-PAR-CLIP is widely used for high-throughput miRNA target characterization. Here, the authors show that the previously neglected non-T-to-C clusters denote functional miRNA binding events, and develop microCLIP, a super learning framework that accurately detects miRNA interactions.

## Introduction

**Fig. 1:**
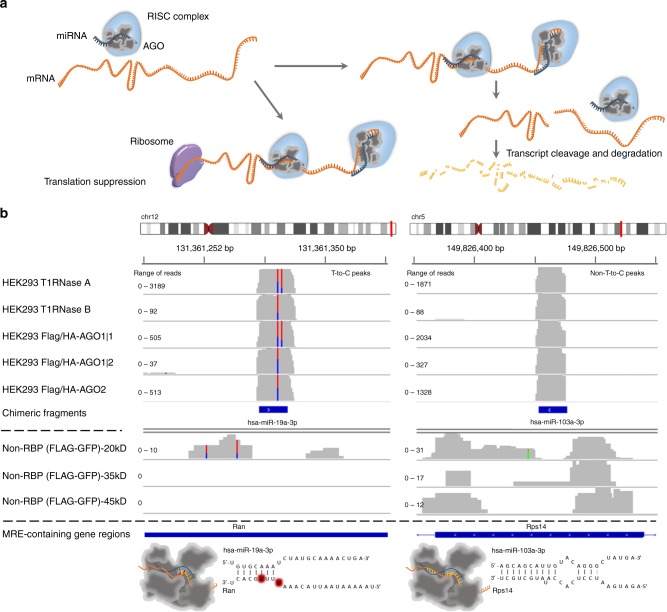
Argonaute crosslinking and immunoprecipitation experiments enable the high-throughput capturing of miRNA targets. **a** Illustration of miRNA targeting. miRNAs are loaded on AGO2 and guide the RISC complex to target MRE(s). RISC binding to its target genes can either cease their translation or induce their cleavage and/or degradation. **b** Peaks derived from 5 AGO-PAR-CLIP libraries on HEK293 cells and from 3 non-RBP background libraries are presented for T-to-C and non-T-to-C AGO-bound regions. The red-and-blue vertical lines represent T-to-C transition sites. Both types of AGO-enriched clusters are clearly distinguished from background signal. Chimeric miRNA-target fragments overlap with (non-)T-to-C peaks providing direct validation for specific miRNA-target pairs (hsa-miR-19a-3p–*Ran* and hsa-miR-103a-3p–*Rps14*). microCLIP identifies the aforementioned interactions as a *7-mer* (*chr12:131,361,200–131,361,400, Ran* gene 3′ UTR) and an *8-mer* with a 3′ compensatory site (*chr5:149,826,350–149,826,550, Rps14* gene CDS), respectively. The 3D depictions of AGO2 were based in the PDB structure 5JS1

Crosslinking and immunoprecipitation sequencing (CLIP-Seq) enabled the high-throughput mapping of RNA-binding protein interactions. microRNAs (miRNAs) are central post-transcriptional gene expression regulators, actively researched for their role in most physiological and pathological conditions, as well as for their potential as biomarkers and/or therapeutic targets^1^. They are small single stranded RNA molecules that are loaded into Argonaute (AGO) to induce target cleavage, degradation, or translational suppression (Fig. 1a). Photoactivatable Ribonucleoside-Enhanced Crosslinking and Immunoprecipitation (PAR-CLIP) variant against AGO proteins is a widely used methodology for miRNA targetome characterization. PAR-CLIP experiments have been performed to map miRNA-gene interactions on a transcriptome-wide scale for healthy or diseased cell types and have provided valuable insights into miRNA regulation of pathogen infections and cancer^2,3^. They are considered among the most powerful high-throughput methods for the characterization of miRNA targets.

During the past few years, computational methods devoted to AGO-PAR-CLIP data analysis have been elaborated by employing different mathematical models and feature sets. MIRZA^4^ implementation employs a biophysical model, while PARma^5^ provides canonical miRNA seed family interactions by processing significantly overrepresented kmers. microMUMMIE^6^ is another state-of-the-art approach based on a six-state hidden Markov model for characterizing the background, the AGO-bound clusters and their flanking regions. Its core algorithm solely processes T-to-C enriched clusters determined by PARalyzer^7^ and recognizes miRNA-binding sites with (im)perfect seed complementarity. These approaches cannot be readily used on sequencing data, since they require extra pre-processing steps and the creation of non-standard file types.

Current algorithms made the complex analysis of AGO-CLIP-Seq datasets accessible to a broader community. However, even these leading implementations present reduced ability to distinguish a large portion of genuine miRNA-targets. To our knowledge, all existing approaches are based on the analysis performed in the seminal paper of Hafner et al.^8^ and depend strongly upon the induced T-to-C conversions (Fig. 1b) to pinpoint miRNA-binding sites.

Aim of the present study is to revisit, identify, and address current obstacles in AGO-CLIP analysis, in order to enable the accurate determination of experimentally supported functional miRNA targets. We propose microCLIP, an in silico framework for CLIP-guided identification of miRNA interactions. microCLIP incorporates novel aspects in PAR-CLIP analysis and increases the experiment’s scope and robustness. Computational approaches for AGO-CLIP-Seq data analysis incorporate machine learning techniques and thus rely heavily on training/validation dataset selection. To this end, we created an extensive experimental collection of miRNA interactions in order to boost the proper optimization of microCLIP algorithm and its exposure to the actual search space complexity.

Our investigation was implemented under a data-driven approach by: (a) creating a comprehensive collection of PAR-CLIP experiments, (b) implementing an extensive compendium of bona fide functional miRNA-binding events from highly specific techniques, and (c) analyzing 123 high-throughput miRNA expression perturbation datasets. This unprecedented list of in-house analyzed experiments enabled us to assess the impact of every algorithmic choice on the accuracy of the provided results.

The most remarkable finding was that clusters depleted on T-to-C conversions, which are always filtered out in such analyses, can aid in the identification of functional miRNA-binding events (Fig. 1b). Importantly, including only T-to-C enhanced cross-linked regions led to a significant loss (60–80%) of the AGO-PAR-CLIP reads across 24 libraries (Supplementary Table 1). We utilized our collection of datasets to assess non-T-to-C peak frequency and functional potential, as well as to identify similarities or differences between these loci and those denoted by T-to-C mutations.

**Fig. 2:**
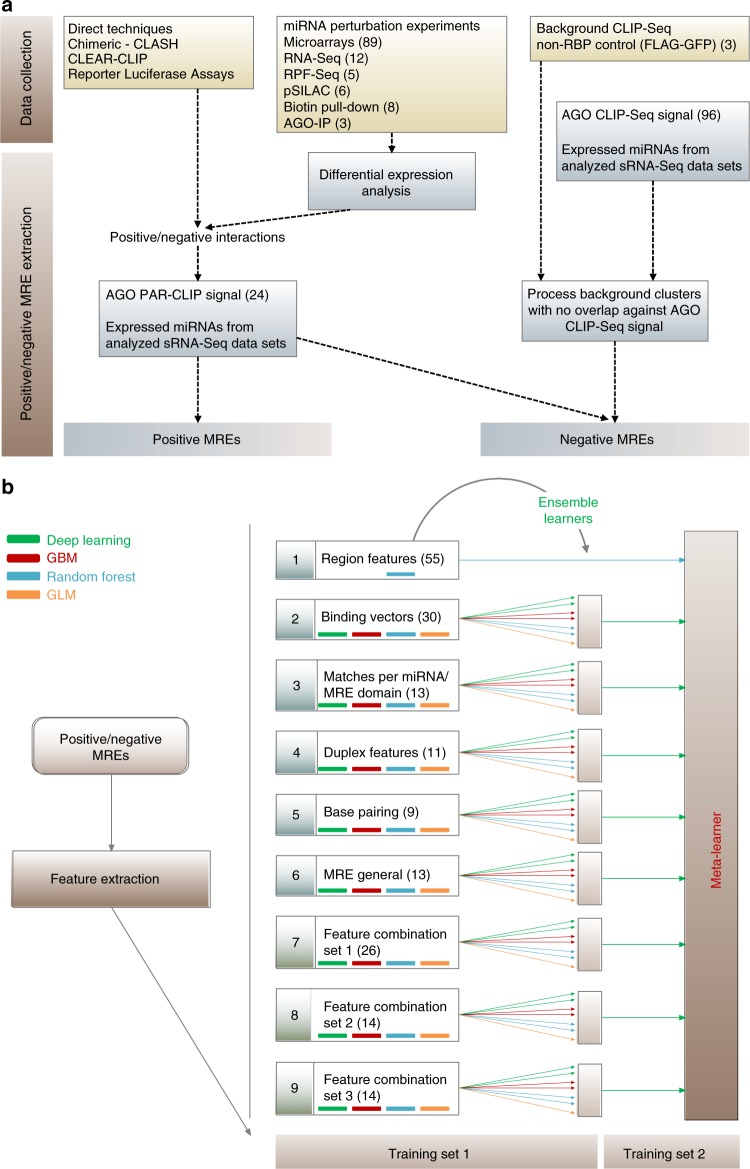
microCLIP in silico framework. **a** Dataset collection and methodology for positive and negative MRE identification. More than 6000 interactions were retrieved from direct techniques and miRNA-target chimeric fragments. Numerous high-throughput experimental data following specific miRNA perturbations enabled the identification of AGO bound or differentially transcribed/translated genes harboring functional binding sites. In order to resolve the exact miRNA-binding sites, positive and negative instances were coupled with signal from 24 AGO-PAR-CLIP libraries. The negative set was enhanced by incorporating background CLIP-Seq clusters. sRNA-Seq datasets were included to determine expressed miRNAs and accurately extract positive/negative MREs. This dataset collection was processed to form the training/test sets of microCLIP deployment (Supplementary Tables 2-3), while 18 miRNA perturbation experiments were segregated (Supplementary Table 6) and introduced in the analyses of Figs. 5–7. **b** Separate subsets of the positive/negative miRNA interactions were used to train the distinct levels of the algorithm’s modeling. 9 base classifiers in the first layer comprise characteristic feature subsets (Supplementary Data 1) that assemble into the GBM meta-learner of the second layer. A super learning scheme is utilized in 8 of the 9 base nodes, weighing outputs from seven individual models. “Region features” node corresponds to an RF classification scheme and consists of CLIP-sequencing-derived features. Five base models (2–6) were designed for MRE-specific features: “Binding Vectors” describe the (un)paired positions along the miRNA/MRE hybrid; “Matches per miRNA/MRE domain” contain attributes of miRNA-target structure and sub-domains; “Duplex Features” include free energy, secondary structure, and AU base pairing features for miRNA and/or target; “Base pairing” encompasses composition descriptors of (un)paired nucleotides; “MRE general” incorporates general MRE-related descriptors. Three supplementary classifiers (“Feature Combination Set 1–3”) comprise unique combinations of features found in base nodes 1–6

microCLIP integrates our findings and provides a robust pipeline for the analysis of all AGO-enriched regions (Fig. 2). It encompasses an approach based on a super learning scheme and employs combinations of deep learning, random forest, and gradient boosting classifiers. The super learner approach was introduced by van der Laan et al. in 2007 and has been shown to be an asymptotically optimal system for machine learning^9^. By using multiple combinations of classifiers, super learning outperforms a single prediction model.

Gene expression regulation has been shown to be highly context-specific at both transcriptional and post-transcriptional levels. In a fashion similar to how transcription factor binding is controlled not only by the site sequence but also by numerous epigenomic mechanisms, microRNA binding and function is dictated by context that can differ significantly among cell types/conditions^10,11^. To this end, we paid specific attention to incorporate data from different cell types and experiments in our study.

microCLIP was trained and evaluated against an extensive set of interactions from hundreds of miRNA specific low/high-throughput experiments across ~50 different cell types. A high quality set, composed of direct miRNA-binding events retrieved from reporter gene assays and chimeric miRNA-target fragments^12–16^, was incorporated in the algorithm’s development and evaluation process (Fig. 2a).

To interrogate the accessibility of miRNA-binding sites residing on (non-)T-to-C clusters and demarcate structural imprints of AGO-bound regions, we examined sequencing data from Parallel Analysis of RNA Structure (PARS) experiments for the first time in such a setting. All enriched regions exhibited strong structural accessibility in the miRNA seed site. non-T-to-C sites were also functionally investigated against 17 gene expression profiling datasets following up/downregulation of individual miRNAs. They proved to harbor functional miRNA-binding events and their incorporation in the analysis revealed an average increase of 14% in identified miRNA-target interactions per PAR-CLIP library.

We subsequently processed an independent AGO-PAR-CLIP dataset in MCF7 cells to evaluate the impact of these neglected sites in downstream analyses. Their inclusion revealed critical pathway components under miRNA regulation that were previously undetected. Pathway members under miRNA control that remained uncovered with conventional pipelines, were validated in miRNA–mRNA expression profiles retrieved from 271 ductal breast cancer samples indexed in The Cancer Genome Atlas (TCGA)^17^.

microCLIP is the first algorithm for AGO-PAR-CLIP data providing more than 80% true positive miRNA-target predictions on a broad test set. Our approach detects 1.6-fold more validated miRNA-target sites when juxtaposed against state-of-the-art implementations, ushering in a new era of miRNA-target annotation. Use of microCLIP can unveil uncharted parts of the miRNA interactome in different physiological/pathological conditions.

## Results

### Α reference collection of bona fide miRNA-binding events

We extracted positive/negative miRNA-target pairs from direct low-yield techniques and miRNA perturbation high-throughput experiments to distinguish AGO-CLIP functional clusters (Methods). Our downstream evaluations included miRNA-binding sites residing on AGO-enriched regions derived from 26 AGO-PAR-CLIP libraries (Supplementary Table 1).

These sets correspond to a comprehensive compilation of miRNA interactions across different experimental methodologies and were utilized as a reference dataset for the examination of important AGO-PAR-CLIP peak properties, as well as for training and evaluation of microCLIP computational framework (Fig. 2a, Supplementary Tables 2–3).

### T-to-C and non-T-to-C PAR-CLIP clusters share common traits

One of the most important steps in PAR-CLIP analysis is the identification of AGO-bound regions for further investigation. This process is based on the presence and percentage of reads harboring T-to-C mutations within a cluster, while all other peaks are omitted from the analysis. We examined if non-T-to-C containing regions, i.e., the major portion of detected clusters, can pinpoint functional miRNA-binding events. Our approach assessed a random set of 4310 and 1700 miRNA-binding sites, supported by T-to-C and non-T-to-C clusters, respectively, located in 3′UTR and CDS regions. More than 65% of miRNA recognition elements (MREs) were derived from direct experimental techniques, while the rest originated from the analyzed miRNA high-throughput perturbation datasets (64 microarray and 12 RNA-Seq experiments) in our reference collection (Supplementary Tables 4–6).

**Fig. 3:**
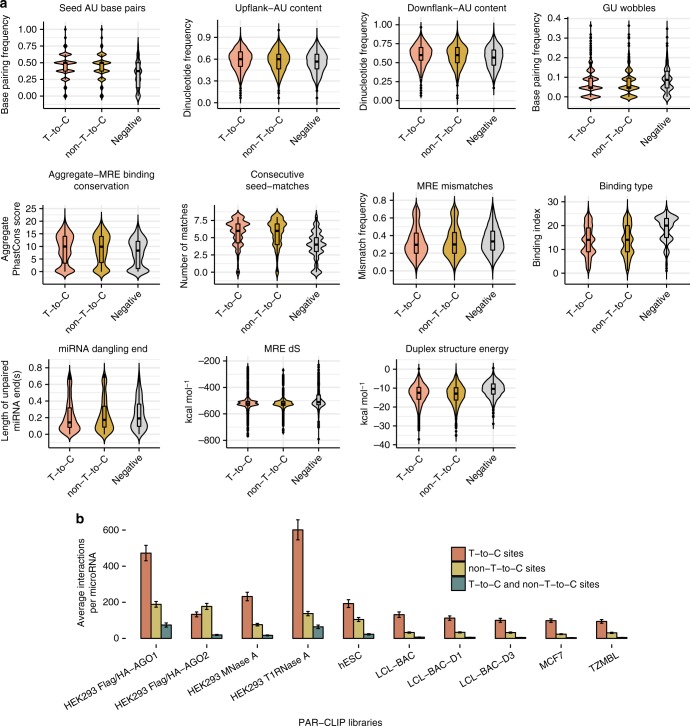
Downstream evaluations of miRNA-binding sites identified on AGO-PAR-CLIP datasets. **a** Distributions of MRE-related features corresponding to positive miRNA interactions in T-to-C and non-T-to-C AGO-bound regions against the relevant densities of negative binding sites. Evaluated descriptors include AU seed base pairs, AU flanking content, GU wobble pairs, MRE conservation, consecutive matches and mismatches per miRNA-target duplex domain, and binding type. The latter feature comprises an extended set of (non-)canonical miRNA base pairings where smaller values indicate stronger seed matches (9mer to 6mer) and greater values correspond to noncanonical and 3′ supplementary sites. Distributions of miRNA-end nucleotides not participating in the hybrid (miRNA dangling end), MRE-related thermodynamic properties of entropy (dS) and minimum free energy are also evaluated. Assessed characteristics of positive miRNA interactions on (non-)T-to-C clusters significantly diverge from respective feature distributions of negative MREs (two-tailed Wilcoxon rank-sum test). **b** Bar plots featuring the average miRNA-target interactions supported by non-T-to-C and/or T-to-C peaks per examined cell type and experimental condition. Mean and standard errors (error bars) of miRNA interactions are shown per library. An average increase of 14% (±8.8%) in the detected interactions was observed across analyzed PAR-CLIP libraries by the incorporation of non-T-to-C clusters

Importantly, we observed that ~28% of the positive MREs, including 1131 chimeric and reporter assay-verified interactions, were exclusively resolved by non-T-to-C AGO-enriched clusters (Fig. 1b). Consequently, our downstream evaluations were initially centered on the comparison of MRE-specific feature distributions between clusters lacking or containing T-to-C sites. We calculated known important attributes for miRNA-target recognition such as the AU flanking content, binding type, matches per miRNA-target duplex domain, minimum free energy, GU wobble pairs, and MRE conservation. Evaluated descriptors of miRNA positive interactions residing on T-to-C clusters significantly diverge from respective densities observed in negative MREs (Fig. 3a, range of *P* values _T-to-C_: 5.9 × 10^−198^–4 × 10^−7^, two-tailed Wilcoxon rank-sum test, *n*_T-to-C_ = 4310, *n*_negative_ = 1423). We show for the first time, that features related to miRNA targeted sites on non-T-to-C clusters also significantly differentiate from relevant estimates corresponding to negative miRNA-target instances (Fig. 3a, range of *P* values _non-T-to-C_: 7.8 × 10^−139^–14 × 10^−5^, two-tailed Wilcoxon rank-sum test, *n*_non-T-to-C_ = 1700, *n*_negative_ = 1423). These findings are consistent with our hypothesis that previously discarded non-T-to-C targeted regions display common characteristics with T-to-C sites regarding properties that are considered decisive for miRNA function.

We additionally realized a primary exploratory study to investigate whether non-T-to-C sites exhibit context specificity as T-to-C sites (Supplementary Note 1). Following the seminal experiment performed in Erhard et al.^10^, we analyzed 10 AGO-PAR-CLIP libraries on virus-infected B-cell lines (Erhard et al.^10^, Skalsky et al.^2^). Both site families exhibited exclusive binding events for a specific context as well as constitutive sites, common across experiments (Supplementary Fig. 1). Context specificity of (non-)T-to-C sites appears to be one more point of concordance between the two site classes. More context-relevant experiments can be included in future related studies, to further validate this initial finding.

### Structural sequencing data unveil accessible AGO-bound loci

**Fig. 4:**
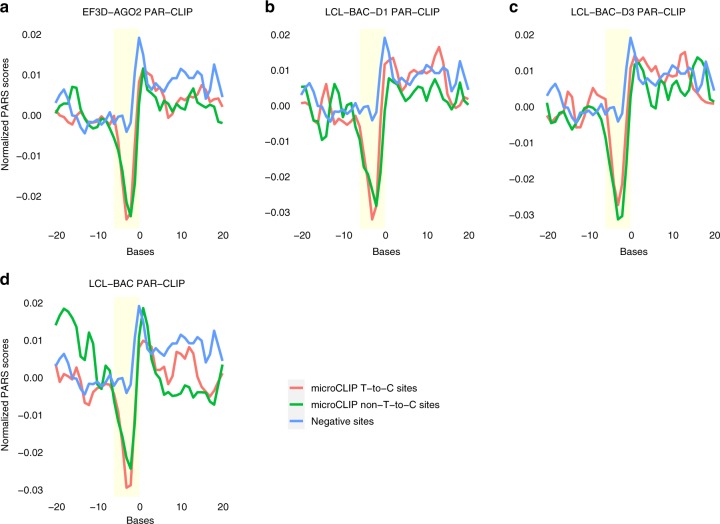
Average PARS scores of AGO-bound regions deduced from the analysis of 4 EBV transformed lymphoblastoid PAR-CLIP libraries. RSS base signals were aligned to the start of the miRNA-target binding site. Base 0 corresponds to the 3′-end of the mRNA, at −1 or −2 nt downstream of the initiation of the direct miRNA seed pairing. Negative PARS scores correspond to single stranded RNA structures, while positive scores to double stranded sites. In the examined AGO-PAR-CLIP EF3D-AGO2 (**a**), LCL-BAC-D1 (**b**), LCL-BAC-D3 (**c**), and LCL-BAC (**d**) datasets, strong structural accessibility occurs in miRNA sites identified on T-to-C (red) and non-T-to-C (green) clusters in the 2–4 nt positions (yellow window) of the miRNA seed pairing. These results significantly differ from respective base scores along negative MREs (light blue) located on AGO-enriched peaks. Statistical significance of position structural changes was calculated using two-tailed Wilcoxon rank-sum test

In order to demarcate RNA Secondary Structures (RSS) of AGO-bound regions compared to a set of negative miRNA sites on mRNA transcripts, we estimated respective PARS scores as introduced by Wan et al.^18^ (Methods). In this approach, AGO-binding efficiency is revealed by RSS signatures observed on mRNA transcripts, since increased structural accessibility is expected in functional conformations. To this end, we investigated whether functional miRNA-target pairs residing on non-T-to-C clusters harbored similar structural properties. We calculated PARS sequencing profiles around AGO-PAR-CLIP-derived miRNA-binding sites in 4 EBV transformed lymphoblastoid cell lines^2^. The analysis of the respective RNase S1 or V1 nuclease signals/intensities at single base resolution enabled the assessment of miRNA site accessibilities in both T-to-C and non-T-to-C clusters. These measurements were juxtaposed against negative MREs comprising miRNAs expressed in the examined lymphoblastoid cell types. The per base averaged PARS scores indicate that strong structural accessibility occurs in the 3′ end of miRNA-target sites and specifically on 2–4 nt positions of the miRNA seed region. These results were identified on interactions residing on (non-)T-to-C clusters and significantly differ from respective base scores along negative MREs located on AGO-enriched peaks (Fig. 4 yellow window; Methods, range of *P* values _T-to-C_: 0.03–3.7 × 10^−5^, *P* values _non-T-to-C_: 0.01–2.4 × 10^−5^, two-tailed Wilcoxon rank-sum test, 3260 < *n*_T-to-C sites_ < 9159, 2119 < *n*_non-T-to-C sites_ < 6473, *n*_negative sites_ = 3059). The outcome of our analysis is consistent with previous observations^19^ and demonstrates that the highest accessibility segregating functional from non-functional binding sites resides towards the initiation of the direct miRNA seed pairing.

### A super learning approach for AGO-PAR-CLIP analysis

We incorporated all the aforementioned observations in an extensive in silico framework. microCLIP is based on ensemble super learning and provides a complete pipeline for experimentally supported miRNA targetome annotation, initiating from aligned (.sam/.bam) PAR-CLIP-sequencing reads. This algorithm, contrary to existing leading implementations, operates on every AGO-enriched cluster, utilizing the previously neglected non-T-to-C clusters. We designed a collection of 131 descriptors associated with CLIP-Seq attributes and miRNA/MRE hybrid derived characteristics (Methods; Supplementary Data 1).

microCLIP adopts a multi-layer super learner classification scheme (Fig. 2b). The first layer incorporates nine different nodes (base classifiers), specialized for subsets of features. Eight of the nine base nodes utilize ensemble deep learning models to weigh the outcomes of seven individual classifiers corresponding to: two Random Forest (RF), one Generalized Linear Model (GLM), two Gradient Boosting Models (GBM), and two Deep Learning (DL) models (Methods). A separate RF base classifier assesses CLIP-Seq derived attributes as well as descriptors linked to MREs and their flanking regions. The training set of the base nodes was composed of 8693 positive and 21,789 negative miRNA-target sites. A separate set of 3276 positive and 6702 negative MREs was included for the development of a GBM meta-learner which aggregates the base classifier outcomes in the second layer of the classification procedure. microCLIP performance was assessed against independent test sets comprising ~5495 instances in total. The composition of respective training/test sets is provided in Supplementary Table 3. miRNA-target pairs were derived from direct low-yield techniques and perturbation experiments described in Supplementary Tables 4–5, 7–9. Training and testing of microCLIP have been performed on independent sets of targeted MRE regions.

### Novel miRNA interactions from AGO-PAR-CLIP clusters

We applied microCLIP and revisited the analysis of 10 public datasets across different experimental conditions (GEO/SRA accessions GSE28859, GSE59944, GSE41437, SRR1045082, SRR359787), in order to explore the extent of miRNA-target pairs that remain uncovered using standard AGO-PAR-CLIP computational approaches. Processed CLIP-Seq libraries were accompanied by RNA-Seq and small RNA-Seq (sRNA-Seq) data to determine the set of expressed transcripts and miRNAs per cell type. By screening every AGO-enriched region, microCLIP reveals a significant portion of targeted genes distinguished only from CLIP clusters presenting no conversion sites. An average 11 ± 6.4% increase of detected targets was observed across the analyzed experiments. Figure 3b summarizes the miRNA-target interactions per library, supported by T-to-C and/or non-T-to-C peaks, respectively. The retrieved results, consistent with our initial inquiry, suggest that the miRNA targetome is not sufficiently covered by inferring targets solely in T-to-C enriched cross-linked regions. The impact of the unrecognized miRNA interactions is also reflected in functional analyses.

### Non-T-to-C miRNA targets disclose functional significance

**Fig. 5:**
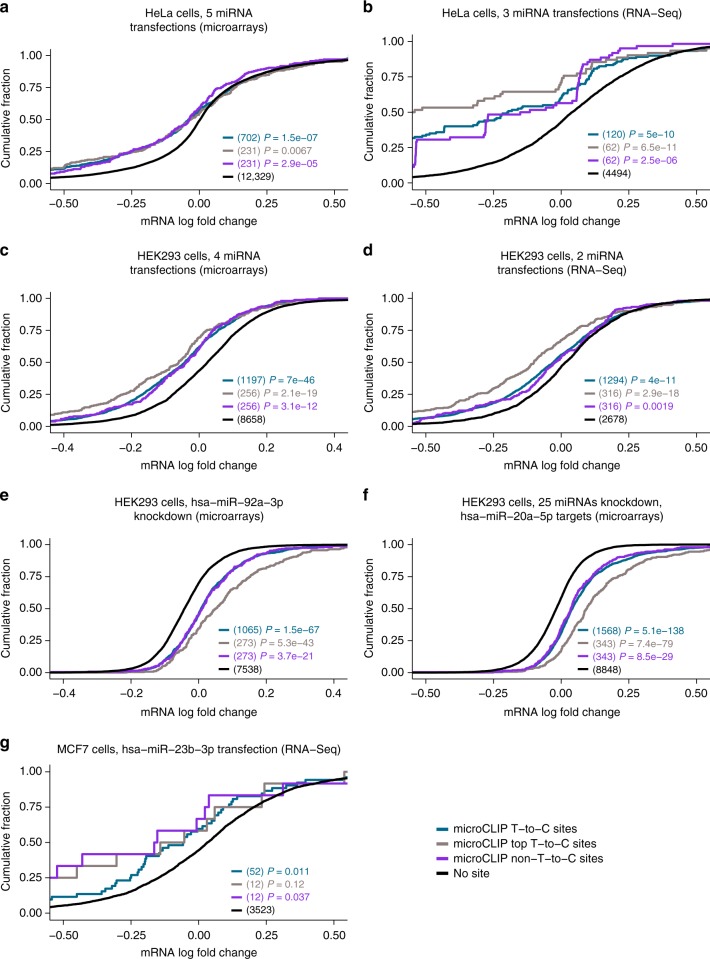
Functional efficacy of microCLIP-detected MREs residing on T-to-C and non-T-to-C AGO-bound enriched regions. miRNA-binding sites were obtained from the analysis of PAR-CLIP libraries (1 HEK293, 1 HeLa and 1 MCF7; GEO accessions: GSM714644, GSM1462574; SRA accession SRA110557) in 3 different cell types. The functional efficiency of predicted targets was examined in 17 public gene expression profiling datasets following miRNA transfection or knockdown (Supplementary Table 6). Response of targeted mRNAs to miRNA perturbation experiments was evaluated independently per tested cell type, experimental technique and conditions (**a**–**g**). Cumulative distributions of mRNA fold changes for targets comprising at least one predicted MRE on T-to-C clusters or supported only by non-T-to-C peaks were compared to those that lack any site of the considered miRNAs. The number of transcripts included in each category is presented in parentheses. Identified targets supported by T-to-C and non-T-to-C clusters exert a significant difference in expression changes compared to transcripts lacking any predicted binding site (two-tailed Wilcoxon rank-sum test). At same numbers of T-to-C and non-T-to-C sites, the former group relates to more responsive targets at miRNA perturbation experiments in **b**–**f**. Fold-change values (log_2_) for perturbation experiments used to evaluate the functional efficacy of MREs supported by T-to-C and non-T-to-C enriched regions are provided in Supplementary Data 2

To investigate the functional importance of miRNA sites residing on AGO-enriched regions presenting insufficient T-to-C substitutions, we utilized 17 public high-throughput gene expression profiling datasets following transfection or knockdown of specific miRNAs (GEO accessions GSE60426, GSE52531, GSE68987, GSE37918, GSE21901, GSE14537, GSE35621, GSE46039, GSE21577, microarrays from the study of Selbach et al.^20^; Supplementary Table 6). These experiments were complemented with AGO-PAR-CLIP datasets conducted in relevant cell types. microCLIP was applied to detect miRNA-gene interactions on HEK293, MCF7 and TZMBL PAR-CLIP libraries (Kishore et al.^21^, Farazi et al.^3^, Whisnant et al.^22^). Response of targeted mRNAs to miRNA deregulation was evaluated independently per tested cell type. In the conducted comparisons, we measured target fold changes in three distinct groups: (i) mRNAs presenting at least one predicted MRE on T-to-C clusters, (ii) mRNAs participating in interactions resolved only by non-T-to-C clusters, (iii) transcripts lacking sites for the examined miRNAs. The distributions of gene expression fold changes in these subgroups are presented in Fig. 5 (expression data available in Supplementary Data 2). In all miRNA perturbation experiments, we observed that detected targets overlapping (non-)T-to-C clusters were significantly downregulated or upregulated upon transfection or knockdown of different miRNAs compared to transcripts having no miRNA-binding site (range of *P* values _T-to-C_: 5.1 × 10^−138^–11 × 10^−3^, *P* values _non-T-to-C_: 8.5 × 10^−29^–37 × 10^−3^, two-tailed Wilcoxon rank-sum test, 51 < *n*_T-to-C_ < 1569, 11 < *n*_non-T-to-C_ < 344, 2677 < *n*_no-site_ < 12,330). Regardless of the perturbation type, T-to-C clusters were observed to relate to more responsive targets at equal numbers of predicted sites (Fig. 5b–f) (range of *P* values _(b-f)_: 2.7 × 10^−11^–3.9 × 10^−2^, two-tailed Wilcoxon rank-sum test, 11 < *n*_T-to-C/non-T-to-C_ < 344). Still, our analysis outcomes confirm our initial assumption that there are functionally important non-T-to-C targets.

The definition of T-to-C locations varies in relevant publications and describes T-to-C loci as those that are covered with reads having at least 5–25% T-to-C substitutions^8,23–26^. For the analyses presented in Figs. 3–5, a minimum 20% T-to-C incorporation ratio defines T-to-C clusters. The selected T-to-C percentage threshold is considered of medium stringency to confidently identify clusters following the experiment’s specifications. The effect of different T-to-C substitution cutoffs (0–20%) on the average of microCLIP-detected miRNA interactions supported by non-T-to-C peaks as well as on the functional efficacy of non-T-to-C supported MREs/targets are presented on Supplementary Figs. 2–3.

### Functional enrichment shows importance of non-T-to-C targets

To demonstrate the ability of detected non-T-to-C interactions to statistically empower downstream analyses, a functional enrichment investigation on KEGG pathways was conducted in highly scored miRNA-target pairs from an independent AGO-PAR-CLIP dataset in MCF7 cells (Farazi et al.^3^).

8921 and 846 unique interactions retrieved from T-to-C and non-T-to-C peaks, respectively, were utilized to form two gene sets: one containing unique T-to-C targets (*n* = 396), and one combining T-to-C and non-T-to-C targets (*n* = 491). A total of 391 genes were common between the two. Pathway analysis of T-to-C targets resulted in 63 significantly enriched terms (*P* < 0.01, one-sided Fisher’s exact test, Benjamini–Hochberg adjustment, 6 < *n*_T-to-C_ < 51), while the combined set yielded 67 enriched terms (*P* < 0.01, one-sided Fisher’s exact test, Benjamini–Hochberg adjustment, 6 < *n*_(non-)T-to-C_ < 58). An average of 2.4 more targets per pathway was observed when non-T-to-C interactions were included.

In both analyses, top-ranking terms were pathways modulating endocrine resistance, growth factor receptor signaling and typical tumor-related processes, like cell growth, migration, and apoptosis. Numerous cancer pathways occupied top positions based on *P*-value scores (Supplementary Fig. 4). This elementary analysis indicated that non-T-to-C peaks assisted in discovering more targeted pathway members.

A case in point is presented for Hippo, TGF-beta and FoxO signaling pathways (details in Supplementary Note 2). In Hippo signaling (hsa04390, *P*_(non-)T-to-C_ = 3.5 × 10^−7^, one-sided Fisher’s exact test, Benjamini–Hochberg adjustment, *n*_(non-)T-to-C_ = 34), among targets derived only from non-T-to-C peaks (Supplementary Fig. 5), miRNA regulation on BIRC5 (Survivin) was notable, since this terminal pathway node is upregulated in breast cancer cells, while its silencing inhibits metastasis and induces apoptosis^27^. non-T-to-C clusters in TGF-beta signaling (hsa04350, *P*_*(*non-)T-to-C_ = 5.42 × 10^−7^, one-sided Fisher’s exact test, Benjamini–Hochberg adjustment, *n*_(non-)T-to-C_ = 23) revealed miRNAs targeting TGIF2, TFDP-1, and EP300, orchestrators of c-Myc and p15 transcription with involvement in cell cycle progression (Supplementary Fig. 5). BCL2L11 (Bim), a terminal FoxO pathway node (hsa04068, *P*_(non-)T-to-C_ = 1.26 × 10^−6^, one-sided Fisher’s exact test, Benjamini–Hochberg adjustment, *n*_(non-)T-to-C_ = 30), established as anti-proliferative and apoptotic marker in breast cancer cells^28^, was revealed to be regulated by non-T-to-C peaks (Supplementary Fig. 6).

To further validate pathway-related interactions from (non-)T-to-C clusters, we investigated miRNA-target expression associations in 271 breast cancer patient samples indexed in TCGA^29^. miRNA and mRNA expression profiles were measured by sRNA-Seq and RNA-Seq experiments. Correlation analysis of expression across samples was conducted for each miRNA-target pair contained in enriched KEGG terms. miRNA-gene expression associations, evaluated separately for interactions resolved by T-to-C and non-T-to-C clusters, are depicted in cumulative distribution plots (Supplementary Fig. 7). The analysis confirmed a significant shift of pathway-related miRNA-target interactions towards more negative correlation coefficients, when compared against a randomly selected subset from all miRNA-gene interacting pairs lacking target sites for the highly expressed miRNAs (*P*_T-to-C_ = 6.7 × 10^−22^, *P*_non-T-to-C_ = 8 × 10^−4^, two-tailed Wilcoxon rank-sum test, *n*_T-to-C_ = 2299, *n*_non-T-to-C_ = 494, *n*_no-site_ = 4000).

The MCF7 dataset case-study exhibited non-T-to-C peaks yielding breast cancer-related interactions that would be lost following standard analysis scenarios. Independent processing of miRNA/mRNA expression profiles from TCGA ductal breast cancer samples clearly distinguishes non-T-to-C miRNA-target pairs from randomly selected pairs lacking target sites for highly expressed miRNAs, validating the previous observations. We conclude that non-T-to-C targets, lost under conventional PAR-CLIP analysis practices, are functionally relevant and should be discerned from experimental noise.

### Evaluation of microCLIP against AGO-CLIP-guided models

**Fig. 6:**
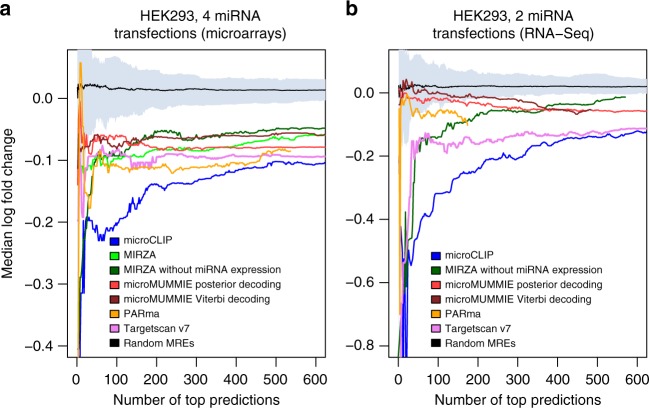
Assessment of microCLIP prediction efficacy against MIRZA, microMUMMIE, PARma, and Targetscan v7. miRNA-target pairs for each AGO-CLIP in silico approach were obtained from the analysis of 7 PAR-CLIP HEK293 libraries and functional investigation was performed by measuring mRNA responses to miRNA perturbations. Unified sets of **a** 4 microarray and **b** 2 RNA-Seq datasets, in which miRNAs were individually transfected into HEK293 cells, were included in the evaluation process. Median fold-change values (log_2_) of the top predicted targets per tested algorithm were plotted and accordingly compared by applying stepwise cutoffs on total predictions. Performed comparisons additionally incorporate a group comprising mean fold changes of 1000 randomly selected genes (without replacement) by using 100 re-samplings. The gray shaded area represents the minimum-to-maximum log_2_ fold-change range of the re-samplings per number of top predictions. microCLIP significantly outperforms all the juxtaposed implementations, detecting targets with the strongest median downregulation, from stringent to loose prediction thresholds (two-tailed Wilcoxon signed-rank test)

To assess microCLIP accuracy and to estimate the information gain with the incorporation of non-T-to-C AGO-enriched regions, we compared our model against MIRZA^4^, microMUMMIE^6^, and PARma^5^. In the evaluation process, AGO-CLIP-guided algorithm performance was also contrasted with Targetscan v7^30^, a state-of-the-art computational approach that defines de novo miRNA-target pairs. Targetscan is a strong seed-based approach and is among the most extensively used target prediction algorithms with higher discriminative power compared to simple seed matching. The performance evaluation was initially accomplished against unified sets of four microarray and two RNA-Seq public datasets in which miRNAs were individually transfected into HEK293 cells (Methods, Supplementary Table 6). An extensive list of interactions for each CLIP-guided program was derived from the analysis of 7 PAR-CLIP HEK293 libraries (Kishore et al.^21^, Memczak et al.^31^; Methods). The retrieved MREs were juxtaposed with deregulated targets identified in the gene expression profiling experiments. To determine the ability of each method to identify the most strongly downregulated targeted genes, detected interactions were ranked according to their provided scores. The median fold changes (log_2_) of the top predicted targets for the different algorithms were subsequently estimated and accordingly compared by applying stepwise thresholds of total predictions. The performance of implementations was additionally evaluated against median fold-change values of randomly selected genes. In the examined miRNA perturbation experiments, microCLIP-detected targets revealed the strongest repression, compared to all the assessed approaches (range of *P* values _microarrays_: 0–8.2 × 10^−74^, *P* values _RNA-Seq_: 0–8.1 × 10^−30^, two-tailed Wilcoxon signed-rank test, 535 < *n*_microarrays_ < 5529, 174 < *n*_RNA-Seq_ < 3129; Fig. 6) and to randomly selected genes (*P*_microarrays_ = 3.3 × 10^−165^, *P*_RNA-Seq_ = 3.3 × 10^−165^, two-tailed Wilcoxon signed-rank test, *n*_microarrays_ = 1000, *n*_RNA-Seq_ = 1000; Fig. 6). microCLIP uncovered interactions with stronger functional impact, when equivalent numbers of top predictions, ordered from highest to lowest scores, were compared. Importantly, the predictions of the tested algorithms were significantly more responsive than expected by chance (range of *P* values _microarrays_: 3.3 × 10^−165^–2 × 10^−89^, *P* values _RNA-Seq_: 3.3 × 10^−165^–1.8 × 10^−30^, two-tailed Wilcoxon signed-rank test, 535 < *n*_microarrays_ < 1001, 174 < *n*_RNA-Seq_ < 1001; Fig. 6).

**Fig. 7:**
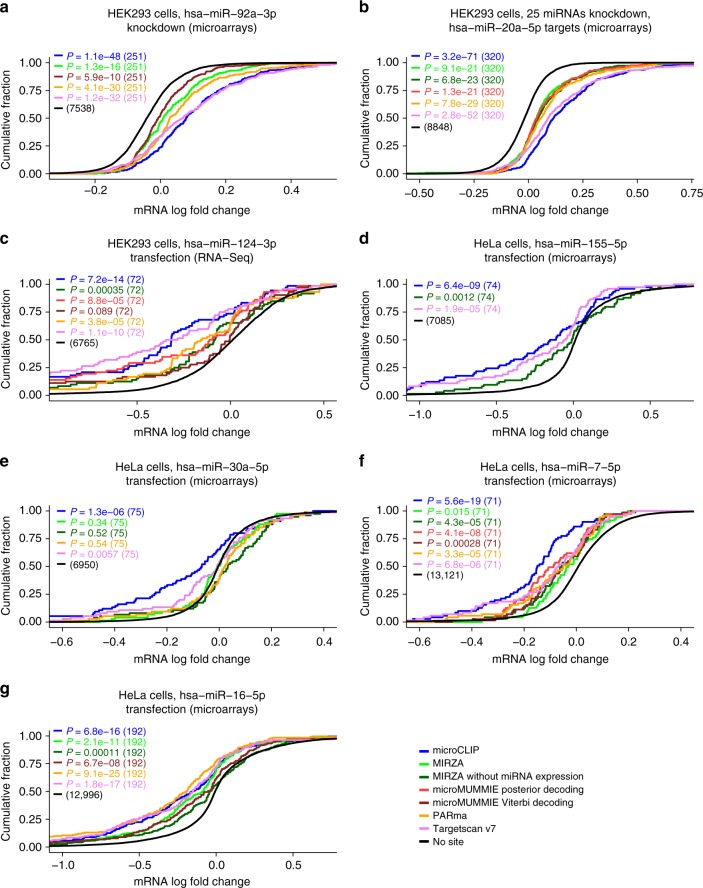
microCLIP performance compared to MIRZA, microMUMMIE, PARma, and Targetscan v7 was examined in seven public gene expression profiling datasets following miRNA transfection or knockdown in HEK293 and HeLa cell lines. miRNA-target interactions for AGO-CLIP in silico approaches were obtained from the analysis of PAR-CLIP HEK293 and HeLa libraries reported in the studies of Kishore et al. and Whisnant et al. Response of targeted mRNAs to miRNA perturbation experiments was evaluated independently per tested cell type, experimental technique and condition (**a**–**g**). Cumulative distributions of mRNA fold changes for targets comprising at least one predicted MRE in the CDS or 3′ UTR regions were compared to those that lacked any site of the considered miRNAs (one-sided Kolmogorov–Smirnov test). Functional efficacy was assessed for equal numbers of top predictions per implementation. Implementations that did not support targets with a fold-change in the examined miRNA perturbation experiments were not included in the relevant cumulative plots. **a**–**f** Identified targets by microCLIP revealed greater site effectiveness than the rest AGO-CLIP-guided implementations. **g** microCLIP performed similarly as PARma and better than the rest of implementations. Targetscan v7 identifies responsive targets, operating on par with in silico approaches based on CLIP data such as PARma, while in **c**, **d** and **g** it displays analogous efficacy as microCLIP. The number of transcripts included in each comparison is denoted in the parentheses. Log_2_-transformed expression fold-change values of all perturbation experiments used in the comparisons are provided in Supplementary Data 2

The performance of microCLIP, MIRZA, microMUMMIE, PARma, and Targetscan v7 was also tested using 3 HEK293 and 4 HeLa expression profiling datasets following miRNA perturbation (Supplementary Data 2, Supplementary Table 6). Interactions were obtained by analyzing HEK293 and HeLa AGO-PAR-CLIP libraries (GEO accessions: GSM714644, GSM1462574) reported in studies by Kishore et al.^21^ and Whisnant et al.^22^, while each miRNA-target pair was characterized by its associated miRNA-binding site with the highest score. To ascertain an impartial evaluation, cumulative distributions of fold changes were compared for equivalent sets of top predicted targets, i.e., genes with one or more predicted MRE, against genes lacking any site(s) for the considered miRNAs. microCLIP exerted significant differences in expression changes compared to transcripts lacking any predicted binding site (range of *P* values _(a–g)_: 3.2 × 10^−71^–1.3 × 10^−6^, one-sided Kolmogorov–Smirnov test, 6764 < *n*_no-site_ < 13,122). Compared to the other CLIP-guided implementations, microCLIP displayed the greatest site effectiveness in most cases (range of *P* values _(a-f)_: 3.1 × 10^−13^–0.031, one-sided Kolmogorov–Smirnov test, 70 < *n* < 321; Fig. 7a–f). In Fig. 7g, it performed similarly as PARma and better than the rest implementations (range of *P* values _(g)_: 0.0005–0.1, one-sided Kolmogorov–Smirnov test, *n* = 192). In this evaluation, Targetscan achieved similar site efficacy as microCLIP in Fig. 7c, d–g. microCLIP demonstrated overall more robust performance compared to this sequence-based predictor (range of *P* values _(a-g)_: 0.002–0.5, one-sided Kolmogorov–Smirnov test, 70 < *n* < 321).

A significant aspect of AGO-CLIP-guided implementations, aside from their ability to detect functionally relevant miRNA interactions, is their efficiency to correctly determine bona fide miRNA-binding sites at a low number of total predictions. Therefore, an extra evaluation was implemented against a validation set of experimentally verified direct miRNA-target pairs to investigate the accuracy of microCLIP-detected interactions compared to existing methods. microCLIP T-to-C model that disregards non-T-to-C site information was also tested (Methods). The utilized validation set is composed of 1674 chimeric and reporter assay-verified interactions from 125 miRNAs (Supplementary Table 3, Supplementary Data 3). The list of predictions for CLIP-guided implementations (Supplementary Data 4) was obtained from an AGO-PAR-CLIP dataset in HEK293 cells (GEO accession GSM714644), while Targetscan (all predictions) and Targetscan conserved predicted sites were utilized. PARma adopts a seed-based approach and identifies miRNA-families with a perfect k-mer match within the PAR-CLIP regions. Accordingly, its predictions have been transformed from miRNA-family sites to miRNA-targeted sites, where every binding region is assigned to each one of the miRNA-family members. The number of correctly predicted MREs per tested in silico method is plotted against the total predictions for different score thresholds (Supplementary Fig. 8a). MIRZA algorithm provides the most probable prediction per cluster. Therefore, an additional evaluation was performed by including only the top scored miRNA-binding site per AGO-peak region, in order to ascertain fairness against all implementations (Supplementary Fig. 8b). Since PARma cannot provide a single top prediction at the miRNA level, all miRNAs bound at a specific site with the same score were considered as top predictions. A separate comparison capturing algorithms’ efficiency to predict correct miRNA-target interactions at different levels of total predictions was also conducted (Supplementary Fig. 8c). The validation set was the same as in the aforementioned evaluations, collapsed into 1527 miRNA-gene interactions. Targetscan operated in the absence of AGO-CLIP data, while predicted interactions of CLIP-guided implementations were defined from PAR-CLIP clusters overlapping full transcript regions. The results demonstrate that although Targetscan methods perform well, in silico approaches based on CLIP data, like microCLIP and PARma, have a significantly better performance. Baseline seed methodologies with and without conservation only identify a small proportion of the MREs presented in the positive test set when they operate on AGO-CLIP enriched regions (Supplementary Fig. 8a, b). microCLIP exhibits a markedly greater ability to discriminate miRNA interactions at equivalent numbers of total predictions, providing a significantly higher sensitivity in the algorithm’s complete predictions set (Supplementary Fig. 8a–c).

## Discussion

miRNA post-transcriptional regulation is critical to numerous mechanisms and therefore is intensively explored with computational approaches and novel experimental techniques. Current limitations of available AGO-CLIP-guided implementations undermine the central position of these experiments in the systematic characterization of miRNA targetome. To this end, we propose microCLIP, a cutting-edge algorithm for the identification of transcriptome-wide functional AGO-occupied clusters and associated miRNA-target pairs. Available in silico approaches process solely T-to-C containing clusters, while the efficacy of neglected interactions remains unknown. By revisiting every step of CLIP-Seq analysis process, we unveiled perspectives that increase the scope and accuracy of AGO-PAR-CLIP experiments. Οur findings and observations were incorporated in microCLIP computational framework.

The backbone of our investigation was a compendium of positive/negative miRNA interactions deduced by analyzing numerous low/high-throughput experiments. It was utilized for training and evaluation of microCLIP algorithm and testing analysis decisions. Our model circumvents pitfalls and limitations of existing implementations dedicated to PAR-CLIP data analysis, with the ability to be generalized to other CLIP-Seq variants. microCLIP is the first relevant implementation to employ the innovative super learner ensemble framework. It is also the only available A-to-Z computational approach for the analysis of AGO-PAR-CLIP data initiating from aligned sequence reads (.sam/.bam files).

Following thorough examination of properties underlying miRNA targeting efficiency, we observed that positive miRNA-binding sites identified on both T-to-C and non-T-to-C clusters significantly diverge from respective descriptors derived from negative MREs (Fig. 3a). On the basis that miRNA interactions resolved from non-T-to-C clusters may encompass functional importance, microCLIP operates on every AGO-enriched cluster, contrary to existing implementations. Notably, the analysis of PAR-CLIP published datasets with microCLIP revealed miRNA-target pairs supported from non-T-to-C peaks, corresponding to a 14% average increase in identified interactions per processed library. The impact of including non-T-to-C clusters was reflected in downstream analyses.

In order to further examine the functional significance of non-T-to-C interactions, we utilized an extensive collection of miRNA perturbation experiments. microCLIP-predicted MREs supported from clusters containing or lacking T-to-C mutations exhibited significantly increased repressive effectiveness compared to transcripts without miRNA-binding sites (Fig. 5). In accordance with previous studies^4,8,10^, the evaluation in Fig. 5 shows that T-to-C supported sites possess strong functionality and that they relate to more responsive targets than non-T-to-C sites at equal numbers of top predictions (Fig. 5b–f).

Structural accessibility of miRNA-binding sites designates true targets and is considered of paramount importance in most prediction algorithms. RNA structural signatures of AGO-bound regions were characterized with PARS sequencing experimental data and assessed for the first time in (non-)T-to-C enriched regions. microCLIP-detected MREs residing on both non-T-to-C and T-to-C clusters, shared strong and common accessibility patterns towards the nucleotides initiating the direct miRNA seed pairing (Fig. 4). Additionally, re-analysis of PAR-CLIP libraries in virally infected B-cell lines denoted agreement between T-to-C and non-T-to-C sites in context specificity (Supplementary Fig. 1). These results significantly segregate functional miRNA interactions from negative MREs residing on AGO-enriched peaks.

An independent pathway enrichment analysis directly showed that miRNA regulation of important signal transduction members may remain elusive using conventional analyses. Enrichment analysis with T-to-C as well as with (non-)T-to-C targets in MCF7 AGO-PAR-CLIP data revealed numerous cancer-related terms to be among the most significantly enriched terms. non-T-to-C targets increased the number of miRNA-controlled genes per pathway, offering more candidate genes for examination, as well as achieving lower statistical significance levels (Supplementary Fig. 4). Pathway-related miRNA-target pairs were additionally subjected to correlation analysis of expression, utilizing (s)RNA-Seq profiles from a cohort of ductal breast cancer samples. The resulting cumulative distributions significantly differentiated from that of remaining miRNA-target pairs lacking MREs for highly expressed miRNAs (Supplementary Fig. 7). Until now, miRNA-gene interactions derived from AGO-bound regions with inadequate T-to-C substitution rates were excluded from the target identification pipeline. This analysis indicates that non-T-to-C targets, accurately retrieved from a robust algorithm, belong to similar biological contexts as targets with sufficient T-to-C substitutions (Supplementary Figs. 5–6).

In-between program evaluations delineated the existence of room for improvement for all algorithms in order to increase accuracy. microCLIP fills the existing gap by detecting interactions with the strongest functional efficacy and with its capacity to correctly identify positive MREs, providing 1.6-fold more validated target sites when juxtaposed against leading implementations.

The increased accuracy of microCLIP in the multifaceted problem of miRNA-target identification can be attributed to the integration of meticulously curated high/low-throughput experimental datasets in an avant-garde super learner framework and to the inclusion of non-T-to-C sites. The comprehensive construction of miRNA interactomes can guide downstream investigations towards the elucidation of unexplored regulatory mechanisms and key components in different biological processes.

## Methods

### Dataset collection

6724 high confidence MREs were retrieved from direct experiments, including reporter gene assay techniques indexed in DIANA-TarBase repository^15,16^, miRNA-chimeras from CLASH (crosslinking, ligation, and sequencing of hybrids)^13^ and CLEAR-CLIP (covalent ligation of endogenous Argonaute-bound RNAs)^14^ experiments, as well as additional miRNA-target chimeric fragments derived from a previous meta-analysis of published AGO-CLIP datasets^12^. In order to quantify miRNA-induced mRNA expression changes and to identify functional binding sites, 101 miRNA perturbation experiments were analyzed (89 microarray and 12 RNA-Seq experiments, Supplementary Tables 4–6). This process enabled the formation of ~3900 and 4000 positive and negative miRNA-target pairs, respectively. A set of five ribosome profiling sequencing (RPF-Seq) libraries after miRNA overexpression, capturing differentially ribosome-bound transcripts, and six pSILAC (quantitative proteomics) experiments were an additional source for detecting more than 5900 miRNA effects at protein expression level (Supplementary Tables 7–8). The inclusion of AGO-IP and biotin pull-down high-throughput experiments upon specific miRNA perturbation yielded ~2600 miRNA-binding events (Supplementary Table 9). The aforementioned miRNA perturbation experiments enabled the detection of deregulated targets without specifying the exact binding sites^15^. miRNA-targeted regions were extracted from AGO-bound enriched regions present in at least 1 of 24 AGO-PAR-CLIP-sequencing libraries (Supplementary Table 1). Published background PAR-CLIP libraries^32^, stably expressing a commonly utilized non-RBP control (FLAG-GFP), were incorporated in our pipeline to identify non-specific AGO-bound transcripts and deduce more than 24,000 negative miRNA-binding sites. A compendium of 96 AGO-CLIP-Seq experiments was derived from DIANA-TarBase and used to further select background-derived MREs displaying no overlap with AGO-enriched regions (Fig. 2a).

### Analysis of high-throughput miRNA perturbation experiments

High-throughput experiments were collected to measure gene expression alterations after specific miRNA transfection, silencing, or knockout. Log_2_ fold-change values as calculated from differential expression analyses of control versus post-treatment state enabled the formation of miRNA–mRNA positive and negative interactions.

A total of 44 microarray studies of distinct experimental conditions (Supplementary Table 4, 6) covering 43 human cell lines and 49 miRNAs were examined to deduce positive and negative miRNA-target interactions. In-house analysis was initiated from microarray raw data (Affymetrix.CEL files). Probe set summarization was implemented using Robust Multi-Array Average (RMA) with R packages affy^33^ or oligo^34^. Annotation of probe sets to Ensembl Gene IDs was accomplished using the chip-specific annotation R packages hgu133a2.db, hgu133plus2.db or hugene10sttranscriptcluster.db. miRNA-treated and control samples in each experiment were analyzed independently of other cell lines or miRNA treatments. Log_2_ fold-change ratios and *p* values were calculated with limma package^35^, following package instructions on Single-Channel Designs. Probe sets mapping to the same gene were averaged to calculate its fold-change. A log_2_ fold-change cutoff of ±1 ( > 1 or < −1, respectively), depending on the type of regulation, was applied to determine negative and positive interaction subsets. For GSE8501 experiment conducted in Rosetta–Merck microarrays, error-weighted log_10_ intensity ratios were retrieved and transformed to log_2_-scale.

Ribosome profiling sequencing (RPF-Seq) and RNA-Seq libraries treated with specific miRNA overexpression, 12 experimental conditions in total (Supplementary Tables 5–7), were retrieved from Eichhorn et al.^36^, Nam et al.^11^, Pellegrino et al.^37^, Zhang et al.^38^. To identify positive/negative miRNA interactions, a ±0.5 log2 fold-change threshold was applied to genes presenting > 10 RPKM expression.

Quantitative proteomics datasets (pSILAC) in HeLa cells following the individual overexpression of five human miRNAs (let-7b, miR-1, miR-16, miR-30a, and miR-155) or knockdown of let-7b (Supplementary Table 8) were derived from Selbach et al.^20^. Positive/negative miRNA interactions were deduced using a ±1 log_2_ fold-change threshold, respectively.

### Analysis of AGO-PAR-CLIP and (s)RNA-Seq expression datasets

AGO-PAR-CLIP datasets from nine studies, corresponding to 13 cell lines in human species, were derived from GEO^7,39^ and DDBJ^40^ repositories (Supplementary Table 1). Fifteen small RNA-Seq and 9 RNA-Seq experiments of similar cell types with PAR-CLIP libraries were analyzed following methodologies as described by Vlachos et al.^41^ to infer expressed miRNAs and transcripts. (s)RNA-Seq datasets were derived from the ENCODE repository and from a series of studies (Supplementary Tables 10, 11). Whole transcriptome depleted from ribosomal RNAs and poly-A selected RNA-Seq libraries were analyzed.

Pre-processing and alignment of PAR-CLIP datasets was realized as described by Vlachos et al.^15^. Initially, libraries were quality checked using FastQC (www.bioinformatics.babraham.ac.uk/projects/fastqc/). Adapter sequences were retrieved from the original publication or GEO/SRA entries, when available. Contaminants were detected utilizing in-house developed algorithms and the Kraken suite^42^. Pre-processing was performed utilizing Cutadapt^43^. PAR-CLIP reads were aligned against human reference genome (GRCh37/hg19) with GMAP/GSNAP^44^ spliced aligner, appropriately parameterized to identify known and novel splice junctions. microRNA expression was quantified using miRDeep2^45^. Ensembl v75^46^ and miRBase v18^47^ were used as annotation for genes and microRNAs, respectively. Top expressed miRNAs and AGO-PAR-CLIP data in each cell type, were jointly utilized as input to microCLIP in silico framework for miRNA-target identification. Specifications on the processed 36 datasets are provided in Supplementary Tables 1, 10, 11.

For the analyses presented in Figs. 3–5 and Supplementary Figs. 4, 7, enriched AGO-CLIP peaks covered with reads having at least 20% cross-linked sites in the same position are defined as T-to-C targeted regions.

### Analysis of PARS experimental data

PARS sequencing data on total RNA isolated from lymphoblastoid cells were obtained from Wan et al. study^18^ (GEO accessions GSM1226157, GSM1226158). The identification of single or double stranded regions, across the human transcriptome, was derived from deeply sequenced RNA fragments generated from RNase S1 or V1 nuclease treatment of GM12878 cells, respectively.

Raw reads of 51nt length, accordingly pre-processed for quality control and contaminant removal, were aligned against human reference genome (GRCh37/hg19) with GSNAP spliced aligner. This analysis resulted in ~130 M uniquely mapped PE-sequenced fragments per sample. In order to derive structural signals in RNase S1 or V1 nuclease experiments at single base resolution, we calculated single(S1) and double(V1) stranded raw reads initiating on each nucleotide. The number of PARS tags per sample starting at each base were normalized by sequencing library depth. These base intensities were subsequently combined into the formula described by Wan et al. to compute PARS scores.

RSS were defined by estimated PARS scores in the vicinity of PAR-CLIP-derived miRNA-binding sites in four lymphoblastoid cell lines from the study of Skalsky et al.^2^. miRNA-mRNA interactions were identified in both T-to-C and non-T-to-C PAR-CLIP clusters, corresponding to transcripts with > 1 TPM expression in GM12878 cells. For expressed miRNAs ( ≥ 50 aligned reads per miRNA) in respective EFD3-AGO2, LCL-BAC, LCL-BAC-D1, and LCL-BAC-D3 EBV infected lymphoblastoid cells, we included collapsed miRNA-binding sites residing within the PAR-CLIP clusters. For the performed comparisons, we incorporated negative MREs extracted from different high-throughput miRNA perturbation experiments (more detailed description in Methods “Analysis of miRNA transfection/knockdown high-throughput experiments”). MREs utilized for the assessment of RSS signatures on AGO-bound clusters and the derivation of (non-)functional conformations of miRNA-target base pairings, were localized on coding and 3′UTR regions. The examined sites had to present S1 and V1 signals in at least half of their occupied bases.

sRNA-Seq and RNA-Seq datasets were retrieved from ENCODE consortium (GEO accession numbers GSM605625, GSM1020026, GSM1020027, GSM1020028, GSM1020029, and GSM1020030).

### microCLIP in silico framework

Feature set description: A set of 131 descriptors (Supplementary Data 1) with non-zero variance was included in microCLIP. The extracted features were retrieved from positive/negative miRNA interactions, identified on AGO-bound locations in different PAR-CLIP datasets. They comprised PAR-CLIP-specific descriptors, such as substitution ratios and distance of conversions from the MRE start, as well as coverage metrics. Aggregate substitution ratios, positions, and distances independent of the transition type were also included. In order to estimate MRE and AGO-peak respective sequencing coverage, we calculated normalized RPKM values for miRNA-target sites and clusters.

Moreover, single base and dinucleotide contents for miRNA-binding and respective flanking regions, complexity features for the MRE and proximal upstream/downstream sequences were introduced to microCLIP model. BLAST’s DUST score^48^ and Shannon-Wiener Index^49^ constituted measurements for masking sequence complexity. Other descriptors were formed to represent energy-related variables for the duplex structure, while metrics capturing sequence content skewness/asymmetry (GC-skew, AT-skew, purine-skew, Ks-skew) and biases of codon usage were added. Entropy, enthalpy, free energy, and melting temperature (Tm) thermodynamic properties were calculated for MRE sequences in R.

miRNA-target hybrids were associated with different descriptors such as the binding type, duplex structure energy calculated with the Vienna package^50^, positions and nucleotide composition of (un)paired nucleotides. Distinct features have been established to model (mis)matches, bulges, loops, and wobble pairs for miRNA-MRE hybrid structure and sub-domains encountered in the duplex. The distinct domains for miRNA sequences, as defined by microCLIP, are: (i) seed region (2–8 positions), (ii) central region (9–12 positions), (iii) 3′ supplementary/compensatory region (13–16 positions), (iv) tail region (17-3′ miRNA end). Similar regions were designated on the MREs based on the miRNA-binding anchors upon duplex formation.

Our approach incorporates conservation of the MRE and upflank/downflank-MRE regions. phastCons pre-computed scores from genome-wide multiple alignments were downloaded from the UCSC repository^51^ in bigwig format and were utilized to deduce respective evolutionary rates. Conservation signals were computed as mean intensities of the phastCons base-wise scores on miRNA targeted regions, as well as their flanking regions. The conservation of the 5′ MRE binding nucleotides was independently modeled. microCLIP integrates additional features corresponding to the location of the MRE within the AGO-enriched cluster and binding length ratios of miRNA and/or target regions.

The applied super learning scheme benefits from the incorporation of the complete array of features, maximizing their contribution through their parallel use in different classification models in every node. The impact of weaker features and classifiers in optimal super learner design and behavior is shown in Supplementary Fig. 9, where microCLIP performance was compared to three different classification schemes using the independent validation set available in Supplementary Data 3.

Description of the algorithm: microCLIP operates on AGO-PAR-CLIP-sequencing reads, requiring a SAM/BAM alignment file and a list of miRNAs as minimum input. It initially seeks for AGO-enriched regions and resolves coverage and observed transitions. A sensitive pipeline is adopted to scan read clusters for putative targeted sites including a wide range of binding types. The algorithm supports an extended set of (non-)canonical matches including 6mer to 9mer, offset 6mer, 3′supplementary and compensatory sites as well as (im)perfect centered bindings. microCLIP extracts features for each candidate MRE and subsequently scores sites through a super learning scheme.

The adopted framework incorporates two distinct levels of classification. The first layer comprises a group of 9 different nodes (base classifiers), which are aggregated in the meta-classifier of the second layer. The learning procedure is decentralized through the distinct nodes and relevant base classifiers that specialize in different subsets of features (Fig. 2b). “Region Features” node comprises CLIP-Seq-derived features, such as RPKM coverage, substitution frequencies, and region-related descriptors, including nucleotide composition, conservation, sequence energy, complexity, content asymmetry, and biases of codon usage. A set of five additional base classifiers were designed for MRE-specific features. Binary binding vectors of miRNA/MRE hybrid were separately incorporated in a base classifier (“Binding Vectors”). Each vector element corresponds to one (un)paired position in the duplex. Matches per miRNA/MRE sub-domain were added to a distinct base classifier introducing a group of 13 features regarding total and consecutive matches in the miRNA-target structure as well as in MRE and miRNA relevant sub-domains. Another base model consists of miRNA-target duplex descriptors (“Duplex Features”) including miRNA-target duplex structure energy, bulges, internal loops, GU wobbles, and AU base pairing features for the specified miRNA and/or target and relevant sub-domains. The “Base pairing” node encompasses composition descriptors (A, T, G, C) of the (un)paired nucleotides. An extra base learner incorporates MRE general descriptors such as the degree of overlap with the respective cluster, conservation of MRE bound nucleotides, MRE location within the cluster, MRE binding type as well as metrics for duplex paired nucleotides content asymmetry/skewness. The latter five base models are dedicated to the determination of genuine miRNA-binding sites. Non-overlapping feature sets from the aforementioned base nodes are combined into three supplementary classifiers also incorporated into microCLIP framework.

Eight of the nine base nodes adopt a super learning scheme that assembles the output of seven individual Random Forest (RF), Generalized Linear Model (GLM), Gradient Boosting Model (GBM), Deep Learning (DL) classifiers (2 RF, 2 GBM, 2 DL, 1 GLM models). The “Region features” is analyzed by an RF classification scheme. The retrieved scores from each node are aggregated in a final GBM meta-classifier.

Model training: The DL models developed for the microCLIP framework adopt a feed-forward multi-layer architecture. The input layers match the respective feature space and values are subsequently propagated within three hidden layers. We utilized a rectifier activation function to retrieve weighted combinations of the inputs transmitted to interconnected neuron units. Dropout regularization was added to achieve model optimization and avoid overfitting. A cross entropy cost-function was selected to adapt weights during the learning process by minimizing the loss. Bernoulli distribution function was used along with cross entropy (log-loss) to model the response variables. The output layer at the end of the network applies a Softmax activation function so that each neuron (predicted class) results in a probabilistic interpretation. The DL network depth, width, and topology, as well as activation functions and learning parameters were modeled with a tuning-in grid search algorithm using H2O^52^ R package. The RF, GBM, GLM learning models were developed, parameterized, and tuned with the caret^53^ and H2O^52^ R packages.

Base classifiers were trained against a collection of 8693 positive and 21,789 negative miRNA interactions (Supplementary Table 3). The final GBM meta-learner that aggregates the base classifier outcomes was trained against an independent dataset comprising 3276 and 6702 positive and negative instances, respectively. Ten-fold cross-validation was performed on the training data to estimate each model’s accuracy and finalize the algorithm’s learning architecture. Distribution of base model scores on positive and negative instances and their respective performance, in terms of sensitivity and specificity in an independent test set of ~4000 instances (Supplementary Table 3), are depicted in Supplementary Fig. 10. The individual performance of internal classifiers (DL, RF, GBM, GLM) in microCLIP base models adopting a super learner approach is shown using the same set in Supplementary Figs. 11, 12. Additionally, the performance of the super learner scheme against Random Forest models was tested (Supplementary Fig. 9). microCLIP training and required computations for model optimizations were multi-threaded.

A microCLIP model adopting the same super learning scheme, including information only from T-to-C enriched sites, microCLIP T-to-C, was also deployed. Clusters from the training set incorporating adequate T-to-C transition sites were selected as input to re-train the super learning classifier. Additional support for the robustness of CLIP-guided super learner classification irrespective of non-T-to-C site inclusion is provided through evaluations of microCLIP T-to-C algorithm performance (Supplementary Fig. 8), as well as of its prediction efficacy (Supplementary Fig. 13) using the same test and datasets as in Fig. 6.

### Expression correlation analysis on (s)RNA-Seq TCGA samples

271 TCGA ductal breast cancer RNA-Seq and sRNA-Seq samples were obtained from Firehose (http://gdac.broadinstitute.org/runs/stddata__2016_01_28). mRNA and miRNA pre-computed expression values were RPM and RPKM units, respectively. In downstream analyses, miRNAs/mRNAs with zero expression value in at least 70% of the samples were filtered out. miRNAs presenting > 10 RPM in > 10% samples were included, based on miRBase criteria for defining a high confidence set^47^. The mRNA set was specified by applying a threshold of more than 1 RPKM in at least 10% of the processed samples. Zero mRNA expression values were replaced by the lowest non-zero RPKM value per sample. This pipeline resulted in a set of 13,346 mRNAs and 322 expressed miRNAs. Pearson correlation coefficient was computed for each miRNA-target pair across samples.

### Functional analysis of AGO-PAR-CLIP-derived miRNA targets

miRNA-target pairs were retrieved from the analysis of MCF7 PAR-CLIP library (Farazi et al.^3^) with microCLIP in silico framework. The 100 most highly expressed miRNAs and their targets in 3′ UTR regions were retained. Gene set enrichment analysis of AGO-PAR-CLIP-detected miRNA targets was performed for KEGG pathways^54^ using the R package limma^35^. Enrichment *P* values were corrected for multiple comparisons using Benjamini–Hochberg false discovery rate and a 0.01 *p*-value threshold was applied. R package Pathview^55^ was used to visualize targeted pathway members in KEGG pathways.

### miRNA interactions from in silico implementations

In order to form a complete list of interactions for MIRZA^4^, microMUMMIE^6^, and PARma^5^ computational approaches, each algorithm was evaluated on a set of 7 PAR-CLIP HEK293 libraries obtained from Kishore et al.^21^ and Memczak et al.^31^ studies (GEO accessions GSM714644, GSM714645, GSM714646, GSM714647, GSM1065667, GSM1065668, GSM1065669, and GSM1065670). The proposed settings for each implementation were retrieved from the relevant publications.

The MIRZA biophysical model was executed in the “noupdate” mode. The algorithm provides an optional parameterization to introduce miRNA expression profiles. We realized two different runs of MIRZA, with and without cell type-specific miRNA expression values that were extracted from the CLIPZ web server (http://www.clipz.unibas.ch). MIRZA input data were 51nt AGO-bound sequences centered on T-to-C sites and mature miRNA sequences of 21nt length as reported in the model’s restrictions. The “target frequency” score was utilized to evaluate the quality of MIRZA-detected sites.

microMUMMIE algorithm was tested in both Viterbi and posterior decoding modes. Following microMUMMIE’s constraints, we utilized PARalyzer v1.5^7^ to define the set of T-to-C AGO-enriched peaks. An extra pre-requisite annotation step to complement PARalyzer detected clusters was implemented with the PARpipe tool (https://github.com/ohlerlab/PARpipe). Derived files, comprising annotated AGO clusters with positions of T-to-C transitions, constituted the input of the microMUMMIE core algorithm. Predictions with signal-to-noise ratio (SNR, generally correlated with sensitivity) equal to 9.95 were retained, while posterior probabilities were utilized for the evaluation of microMUMMIE’s performance.

PARma was applied on AGO-PAR-CLIP aligned data that were prepared following the algorithm’s described format. The required input files contained genomic locations of aligned CLIP reads along with positions of observed conversion sites. PARma predictions are coupled with Cscore and MAscore scores for the cluster and miRNA seed family activity, respectively. The latter score was utilized for PARma-detected miRNA-target sites evaluation.

Precompiled (non)conserved miRNA site context++ scores for representative transcripts were downloaded from the Targetscan v7.2 site (http://www.targetscan.org/cgi-bin/targetscan/data_download.vert72.cgi). Targetscan v7 algorithm was additionally executed following the proposed settings in order to cover a greater transcript collection, as well as the whole spectrum of Targetscan-detected interactions including 6mer sites. Gene annotation files were retrieved from the Targetscan v7.2 official download page, and the miRNA seed sequence file that is a pre-requisite for the execution of the model was provided by Targetscan developers. The local Targetscan run complements the precompiled data with miRNA-target interactions on transcripts presenting the longest 3′UTR, in cases they are not deposited on the online repository.

### Median fold changes

The comparison between microCLIP and existing implementations was performed using six gene expression profiling datasets following individual transfection of highly expressed miRNAs into HEK293 cells (GEO accessions GSE60426, GSE52531, GSE21901, GSE14537, GSE35621, Supplementary Table 6). Genes with unchanged expression levels (zero log_2_ fold change) following miRNA transfection and/or knockdown have been filtered out. Subsequent measurements were realized at the gene level. miRNA-gene interactions retrieved from each implementation were sorted according to their prediction scores. Each miRNA-target pair was characterized by the highest scored miRNA-binding site overlapping coding or 3′UTR exons, since utilized algorithms provided MRE-oriented prediction scores. In cases of multiple transcript-gene associations, the transcript with the longest 3′UTR was selected. Median expression log_2_ fold changes were estimated in consistence with the number of top predicted targets. Aggregated expression changes of genes were calculated by applying stepwise score thresholds. Paired comparisons required tested programs to have targets at every computational cutoff. Lower mean log_2_ fold changes correspond to stronger downregulation of the detected targets upon miRNA transfection. The statistical differences in the mean log_2_ fold-change values obtained by each implementation were assessed using two-tailed Wilcoxon signed-rank test. Identified targets by each algorithm were also juxtaposed against averaged log fold changes of 1000 randomly selected genes (without replacement). The mean log_2_ fold-change values of the randomly selected genes in different stepwise thresholds were taken and the median curve derived from these values was calculated. Genes with zero fold-change indication were filtered out from the random selection process.

### Statistics

Enrichment analyses were performed using one-sided Fisher’s exact test. Correlations between quantitative parameters were assessed by calculating Pearson’s correlation coefficient. Comparisons between two or more groups were conducted using Wilcoxon’s rank-sum test and Kruskal-Wallis’ test, respectively. In the latter, Wilcoxon’s rank-sum test was performed as a post hoc test in order to assess between-group differences. The one-sided Kolmogorov–Smirnov test was used to test for greater functional efficacy. In cases of multiple hypothesis testing, Benjamini–Hochberg’s false discovery rate was applied to control family-wise error rate. *P* values < 0.05 were considered as statistically significant.

## Electronic supplementary material

Supplementary Information

Description of Additional Supplementary Files

Supplementary Data 1

Supplementary Data 2

Supplementary Data 3

Supplementary Data 4

## Data Availability

The source code for microCLIP framework as well as data and scripts for the evaluation of in silico implementations are available at www.microrna.gr/microCLIP.
