# Supplementary material for: microCLIP super learning framework uncovers functional transcriptome-wide miRNA interactions
Source: Nat Commun. 2018 Sep 6;9:3601. doi: 10.1038/s41467-018-06046-y (PMC6127135; doi:10.1038/s41467-018-06046-y)
Supplement: Supplementary file 1 — Supplementary Information [file 41467_2018_6046_MOESM1_ESM.pdf]

Supplementary Information:

microCLIP super learning framework uncovers functional transcriptome-wide miRNA interactions

Paraskevopoulou *et al.*

Supplementary Figure 1

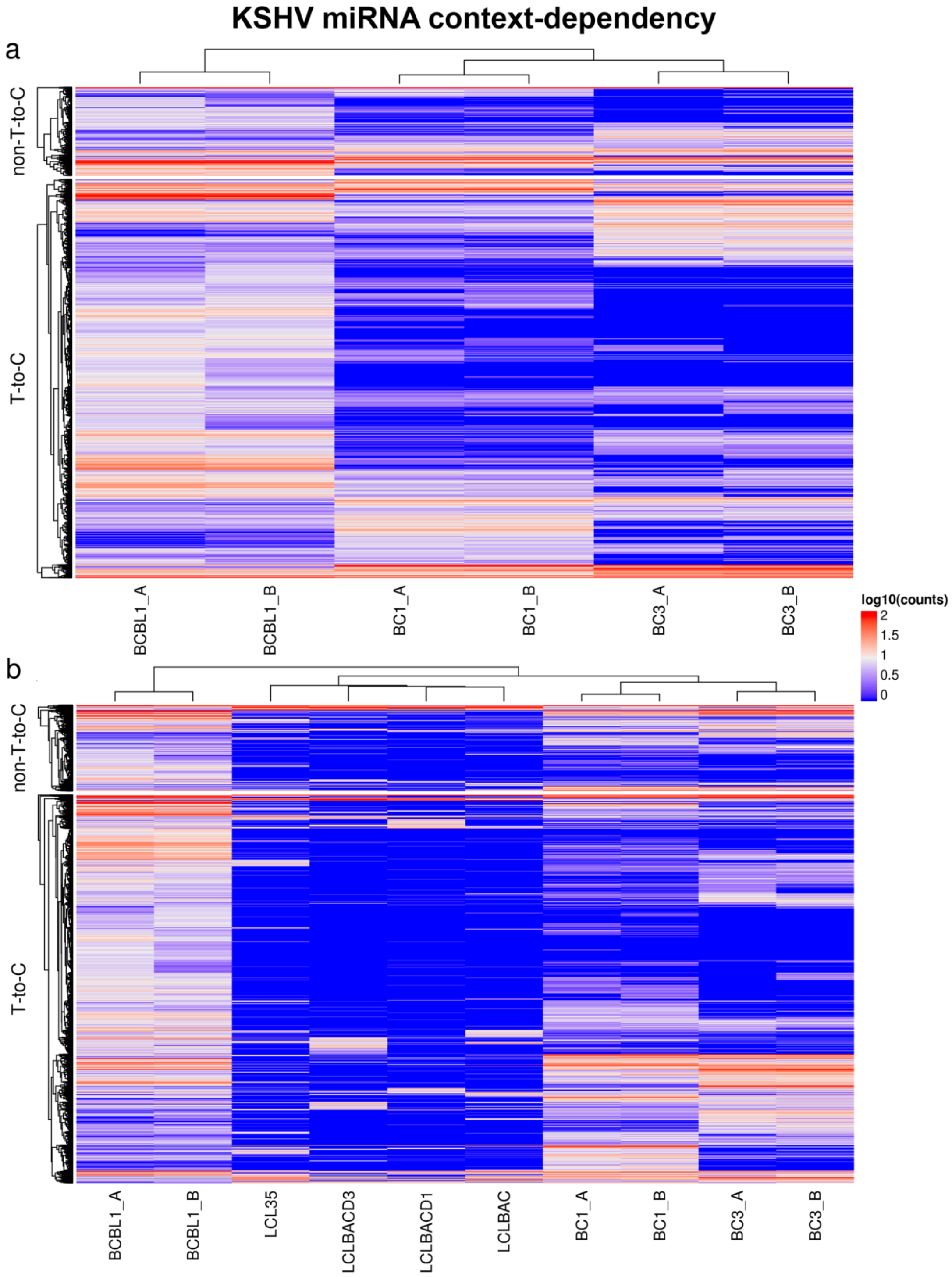

Investigation of KSHV miRNA context-dependency. (a) Heatmap representation of KSHV miRNA (non-)T-to-C targeted regions on BCBL1, BC1 and BC3 B-cell lines. PAR-CLIP enriched regions in protein coding transcripts targeted by KSHV microRNAs and presenting >= 5 normalized reads in at least one library were

analyzed. The 10,474 T-to-C and 2,323 non-T-to-C regions are mutually exclusive. Targeted regions between replicated PAR-CLIP libraries show high reproducibility, while comparison across B-cell lines reveals less overlap. (b) Inclusion of 4 PAR-CLIP lymphoblastoid EBV infected datasets (LCL-BAC, LCL-BAC-D1, LCL-BAC-D3 and LCL35) in the same viral miRNA targeted regions as in (a). Lymphoblastoid libraries present no correlation and significantly less enrichment on most of the KSHV miRNA (non-)T-to-C sites. Notably, similar patterns are revealed between the two site classes.

Supplementary Figure 2

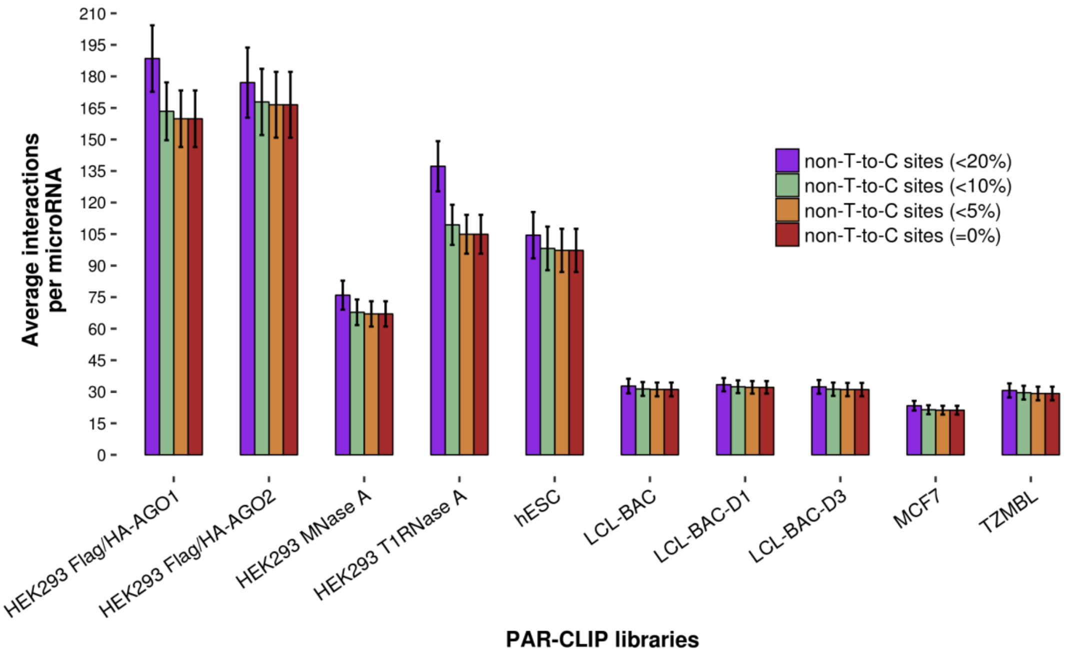

Bar plots featuring the average miRNA-target interactions supported by non-T-to-C peaks for different T-to-C incorporation thresholds (0-20%). Results per examined cell type and experimental condition are displayed separately. Mean and standard errors (error bars) of miRNA interactions are shown per library. An average increase of 13-14% (± (8.2-8.8%)) was observed in the detected interactions for the different cutoffs across analyzed PAR-CLIP libraries by the incorporation of non-T-to-C clusters.

Supplementary Figure 3

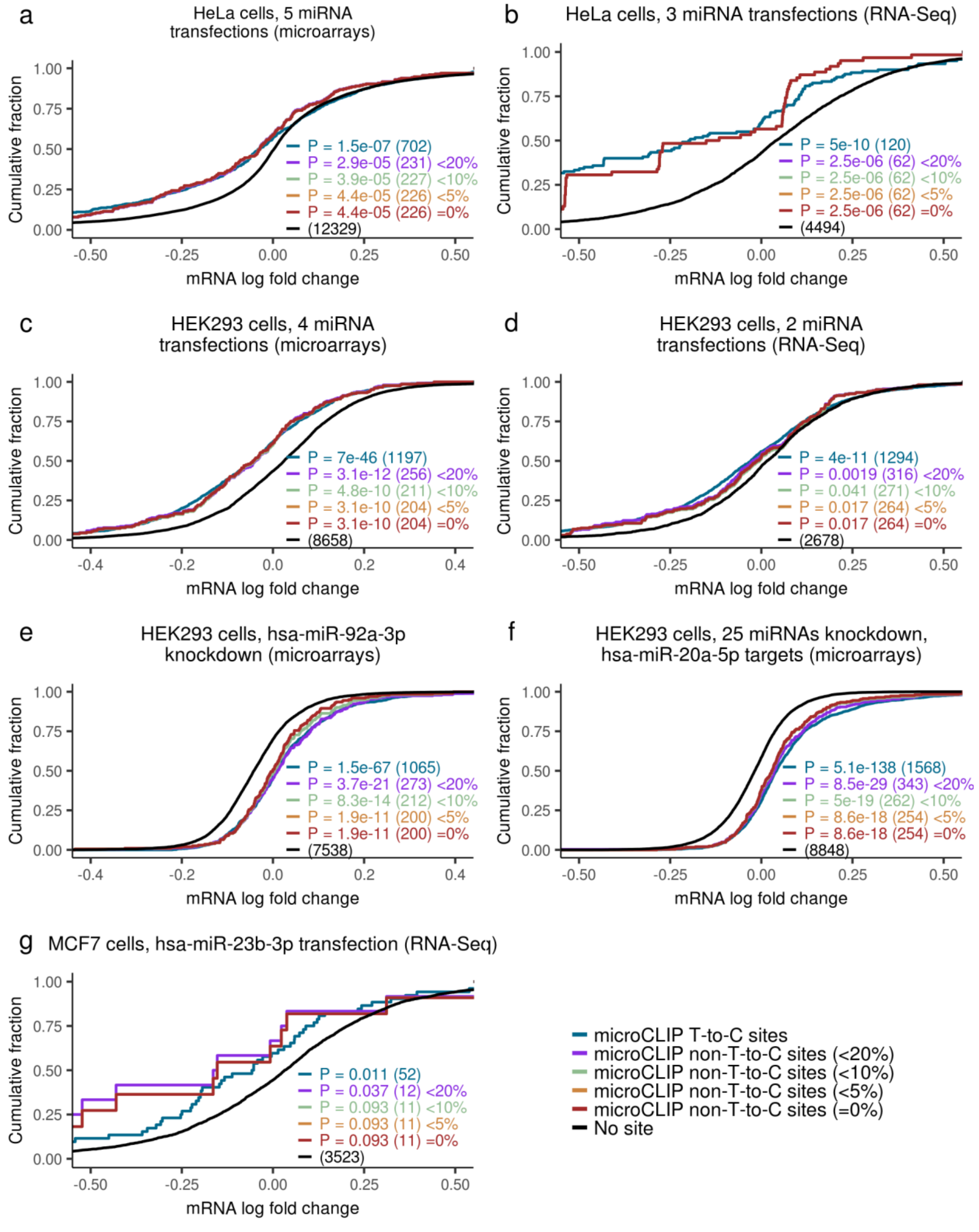

Functional efficacy of microCLIP-detected MREs residing on non-T-to-C AGO-bound enriched regions, upon applying different cutoffs (0-20%) in the T-to-C incorporation. miRNA-target interactions were obtained from the analysis of AGO-PAR-CLIP HEK293 and HeLa libraries reported in the studies of Kishore *et al*. and Whisnant *et al*. The functional efficiency of predicted targets was examined in 17 public gene expression profiling datasets following miRNA transfection or

knockdown in HEK293 and HeLa cell lines. Cumulative distributions of mRNA fold changes for targets comprising at least one predicted MRE on T-to-C clusters or supported only by non-T-to-C peaks were compared to those that lack any site of the considered miRNAs (a-g). The number of transcripts included in each category is presented in parentheses. Identified targets supported by non-T-to-C clusters, examined in any T-to-C incorporation percentage (0%, 5%, 10%, 20%), exert significant differences in expression changes compared to transcripts lacking any predicted binding site (a-f comparisons; two-tailed Wilcoxon rank-sum test). In (g), the small number of exclusively non-T-to-C targets had a negative impact in the achieved statistical power for the performed tests. Log2-transformed expression fold-change values of all perturbation experiments used in the comparisons are provided in Supplementary Data 2.

Supplementary Figure 4

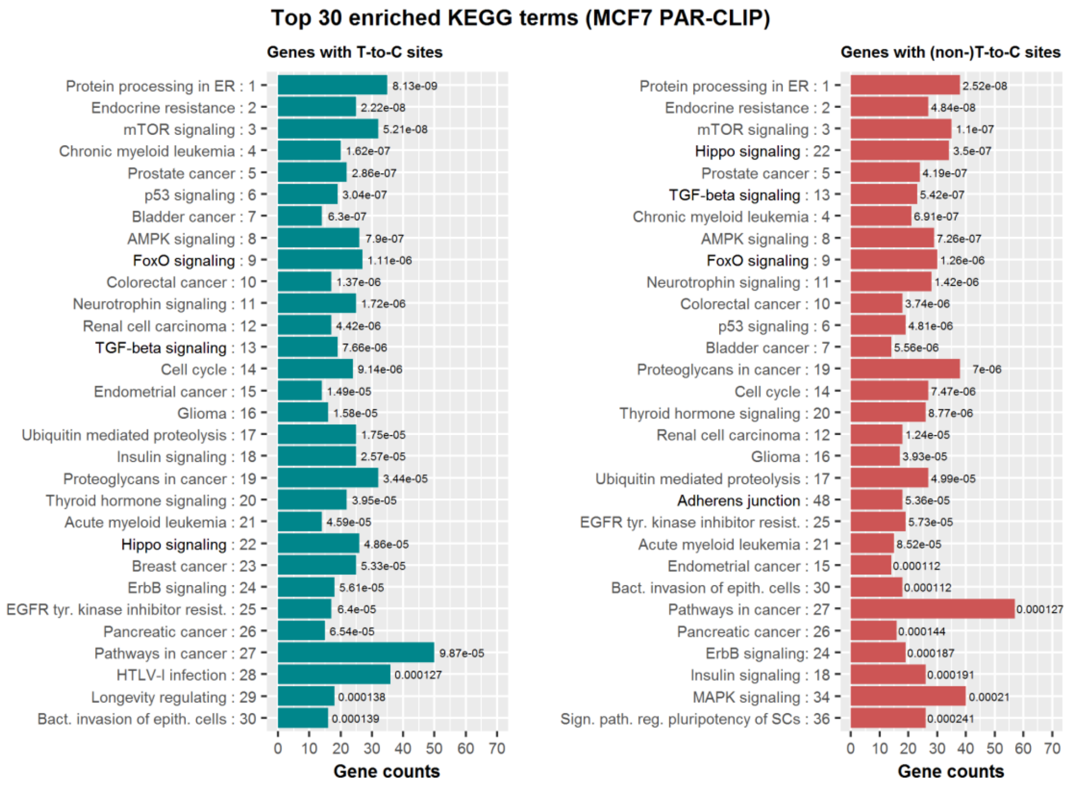

Functional significance of (non-)T-to-C sites in MCF7 AGO-PAR-CLIP dataset. Top 30 KEGG pathways enriched by T-to-C or (non-)T-to-C (combined T-to-C and non-T-to-C) peak containing genes. X-axis depicts number of genes enriching each term. Pathways are ranked according to the enrichment *P* value shown at the end of each bar ($P < 0.01$, Fisher's exact test, Benjamini-Hochberg adjustment, $6 < n_{T\text{-to-}C} < 51$, $6 < n_{(non\text{-})T\text{-to-}C} < 58$). The T-to-C site enrichment rank is provided after pathway description to facilitate comparison with gene set of (non-)T-to-C sites.

Supplementary Figure 5

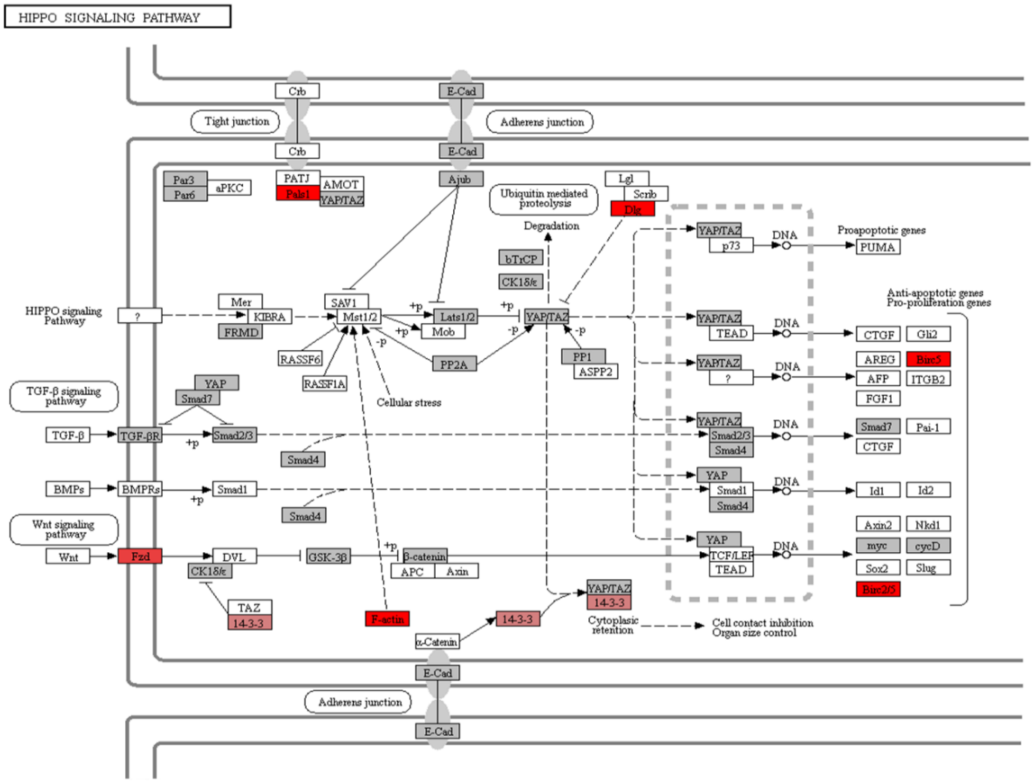

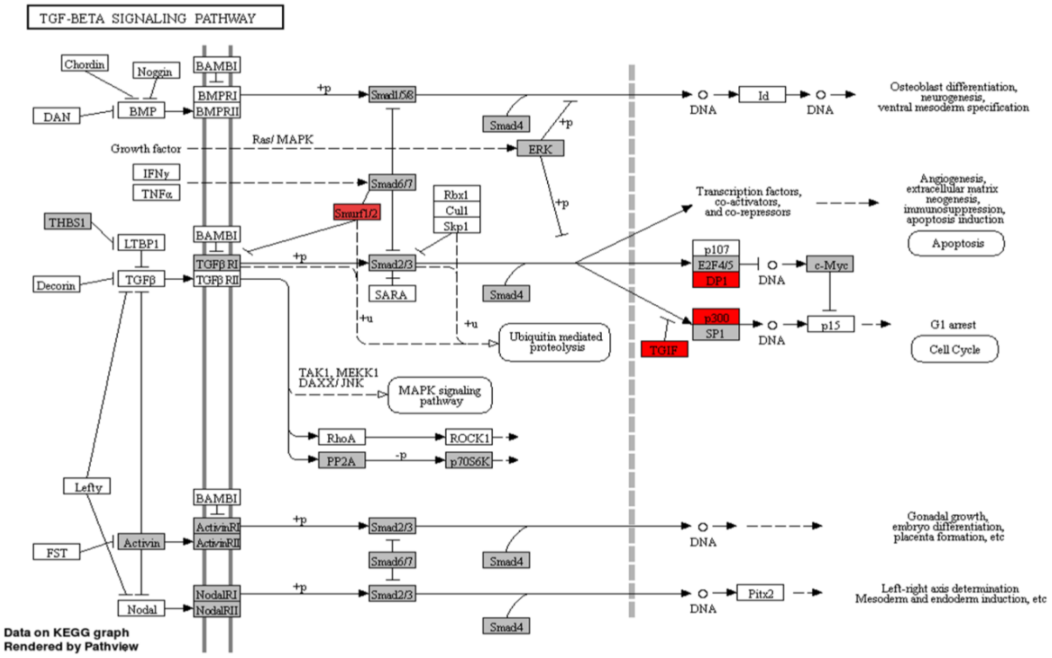

Participation of AGO-PAR-CLIP identified miRNA targets in Hippo and TGF-beta signaling pathways (map numbers hsa04390 and hsa04350 respectively) in MCF7 cells. On each KEGG pathway map, a gradient spanning from grey to red is utilized to highlight miRNA targets revealed from T-to-C and non-T-to-C PAR-CLIP peaks.

Nodes with lighter red tint contain genes found to be targeted by T-to-C peaks and also others by non-T-to-C peaks, e.g. node 14-3-3 in Hippo signaling pathway contains T-to-C targets YWHAH and YWHAZ as well as non-T-to-C target YWHAE.

Supplementary Figure 6

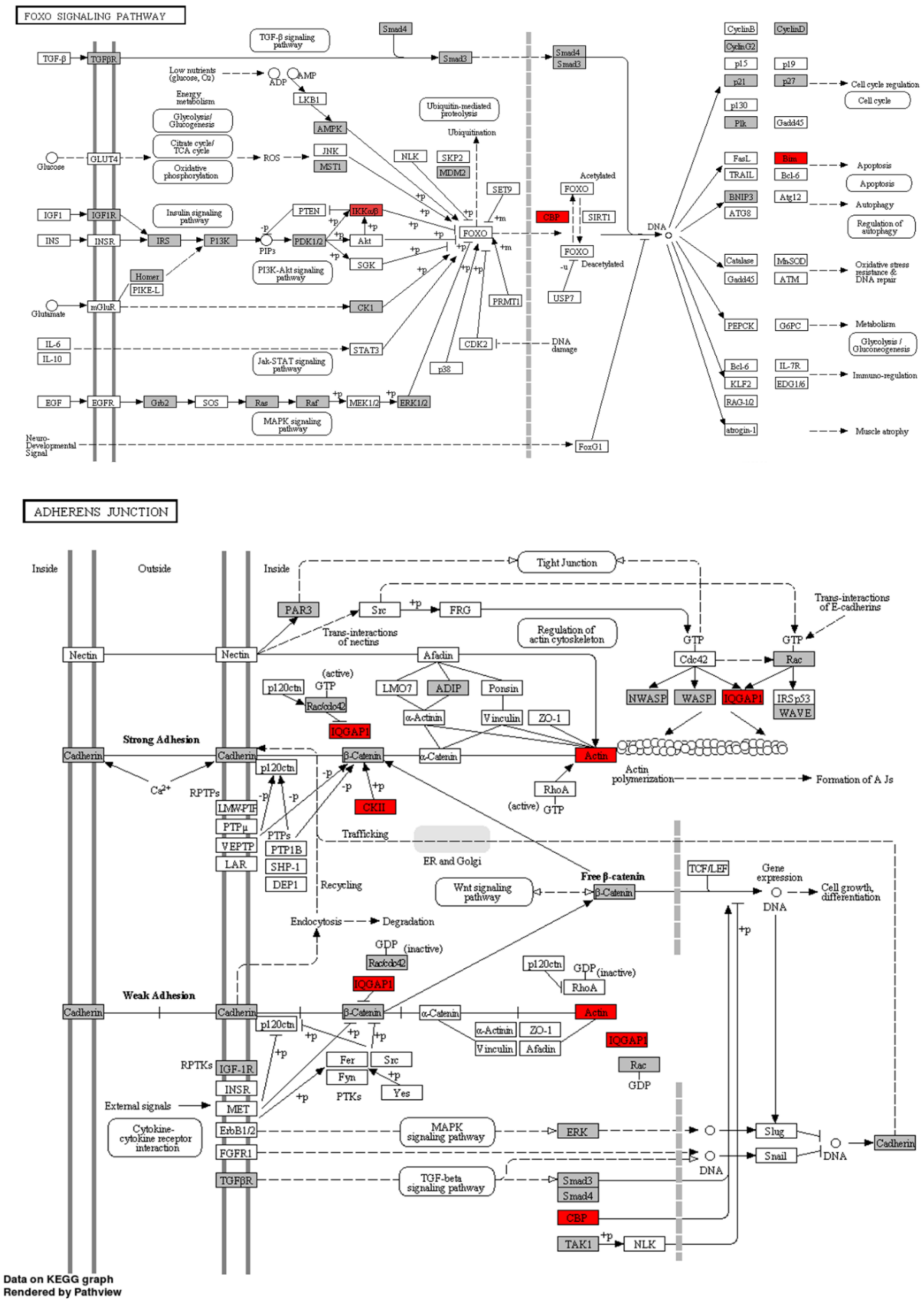

miRNA targets from AGO-PAR-CLIP peaks of MCF7 data set enriching FoxO signaling pathway (hsa04068) and Adherens junction (hsa04520). Grey and red nodes contain T-to-C and non-T-to-C identified targets respectively.

Supplementary Figure 7

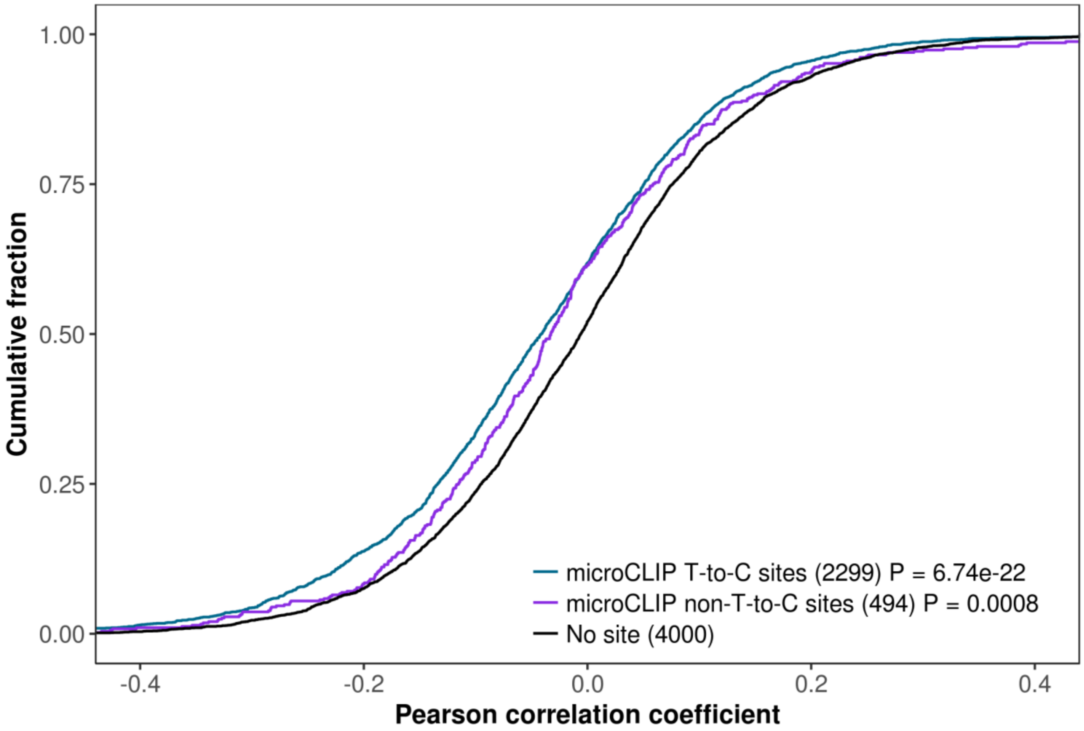

Correlation analysis of expression of pathway-related miRNA-target interactions across 271 TCGA ductal breast cancer samples (patients). Cumulative distributions of miRNA-target expression relationships, evaluated for interactions supported by T-to-C or non-T-to-C AGO-bound regions were compared to a randomly selected set from all the remaining miRNA-gene interacting pairs lacking any target site of the highly expressed miRNAs. The number of genes considered in each category is presented in parentheses. Pathway-related miRNA-target interactions supported by T-to-C and non-T-to-C clusters reveal a significant shift towards more negative correlation coefficient values compared to the no-site distribution (two tailed Wilcoxon rank-sum test).

Supplementary Figure 8

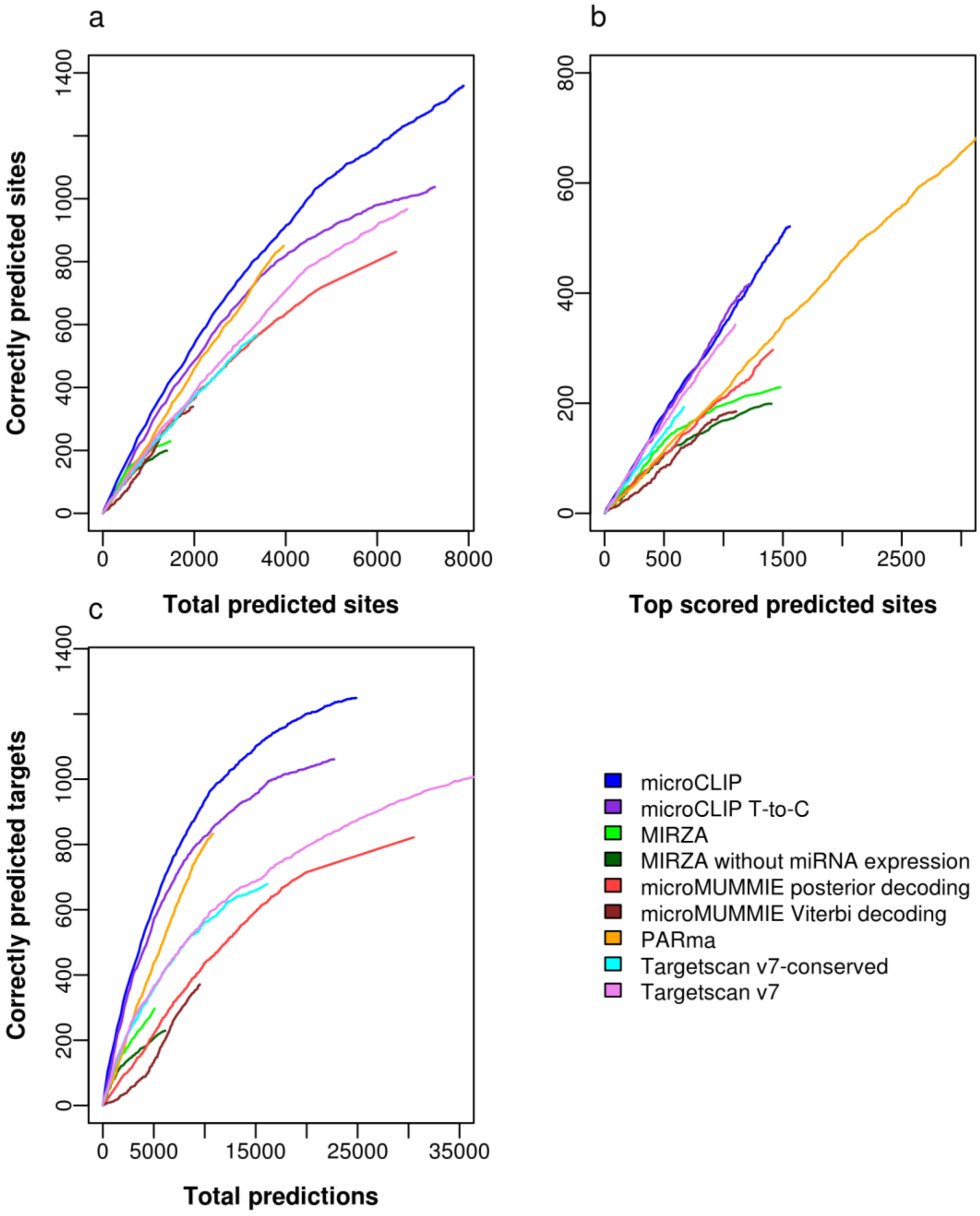

Evaluation of microCLIP performance against microCLIP T-to-C, MIRZA, microMUMMIE, PARma, Targetscan v7 (all predictions) and Targetscan v7 conserved predicted sites. The utilized validation set comprised 1,674 positive miRNA binding sites of 125 miRNAs, derived from chimeric miRNA-target fragments and direct miRNA bindings supported by Reporter Gene Assays. The number of correctly predicted miRNA binding sites for each implementation is plotted versus (a) the total retrieved predictions, (b) the top scored miRNA binding site per AGO-bound enriched region. In (a) and (b) comparisons, we restrict each program's predictions on PAR-CLIP clusters overlapping the validation test set. A

separate comparison (c) captures algorithms' efficiency to predict correct miRNA-target interactions at different levels of total predictions. The validation set is the same as in (a-b) evaluations, collapsed into 1,527 miRNA-gene interactions. For the latter comparison, seed-baseline methods were operating in the absence of AGO-CLIP data, while CLIP-guided implementations on PAR-CLIP clusters overlapping full transcript regions. Predictions derived from CLIP-guided approaches are provided in Supplementary Data 4.

Supplementary Figure 9

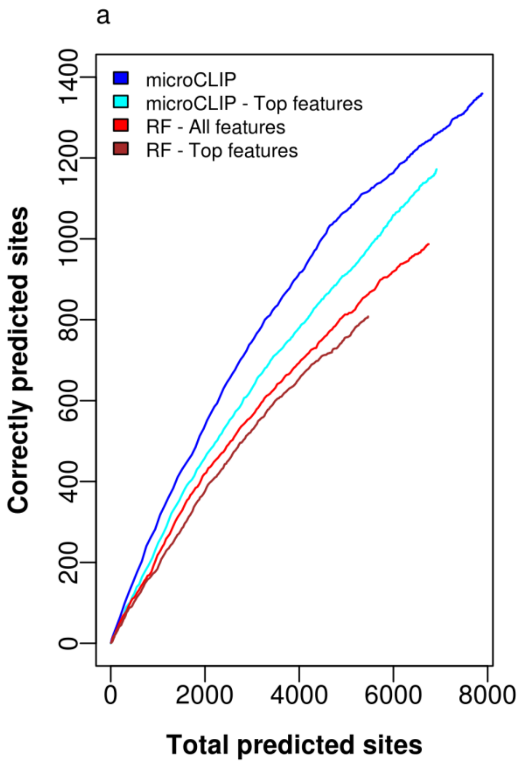

Evaluation of microCLIP performance against 3 alternative classification approaches: a Random Forest classifier comprising all the features; a Random Forest classifier including the top 27 discriminative features (AUC ≥ 65%); microCLIP super learner classification scheme including top performing features per base node (70 descriptors in total, AUC ≥ 65%). The utilized validation set comprised 1,674 positive miRNA binding sites, derived from experimentally validated direct miRNA interactions. (a) The number of correctly predicted miRNA binding sites for each classification approach is plotted versus the total retrieved predicted sites. (b) A separate comparison captures the models' efficiency to predict correct miRNA-target interactions at different levels of total predictions. The validation set is the same as in (a) collapsed into 1,527 miRNA-gene interactions.

Supplementary Figure 10

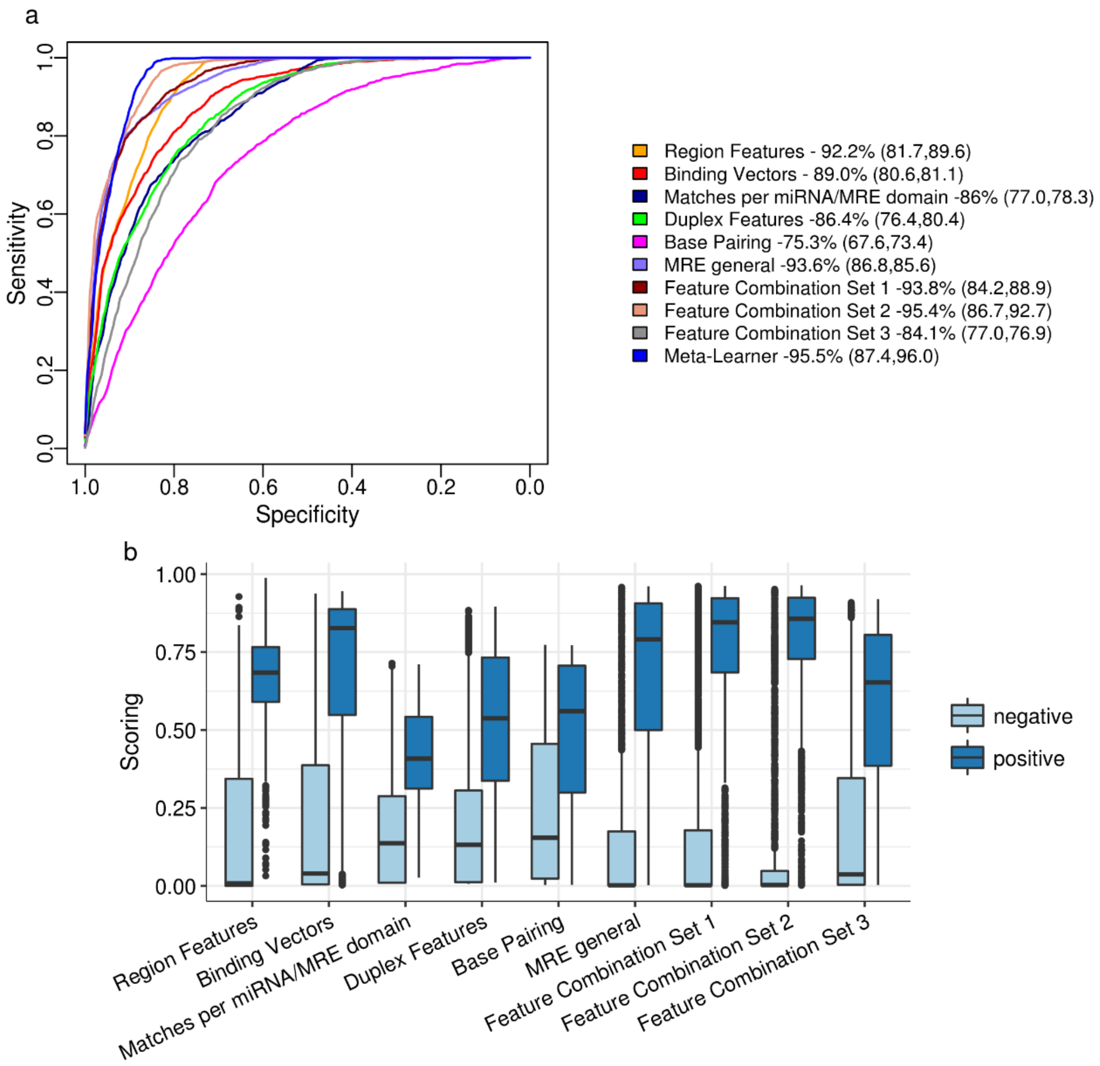

Evaluation of the accuracy of the 9 base model classifiers. Five-fold cross-validation has been implemented on a separate set of approximately 4,000 instances to test the performance of each node. a) ROC curve of each base model displays the classification of positive/negative miRNA binding sites. All the classifiers achieved high performance in a range of sensitivity 73.4% - 92.7% and specificity 67.6% – 86.8% (range of AUC: 75.3% – 95.4%). Their aggregated outcome in the meta-learner of microCLIP framework is provided in a separate curve and exhibits the highest performance in terms of sensitivity and specificity (sensitivity: 96.0, specificity: 87.4, AUC: 95.5%). b) Distribution of base model scores estimated on positive/negative instances of the test set. Whiskers extend up to 1.5*IQR from the lower and upper quartiles (i.e. Q1 – 1.5*IQR, Q3 + 1.5*IQR).

Supplementary Figure 11

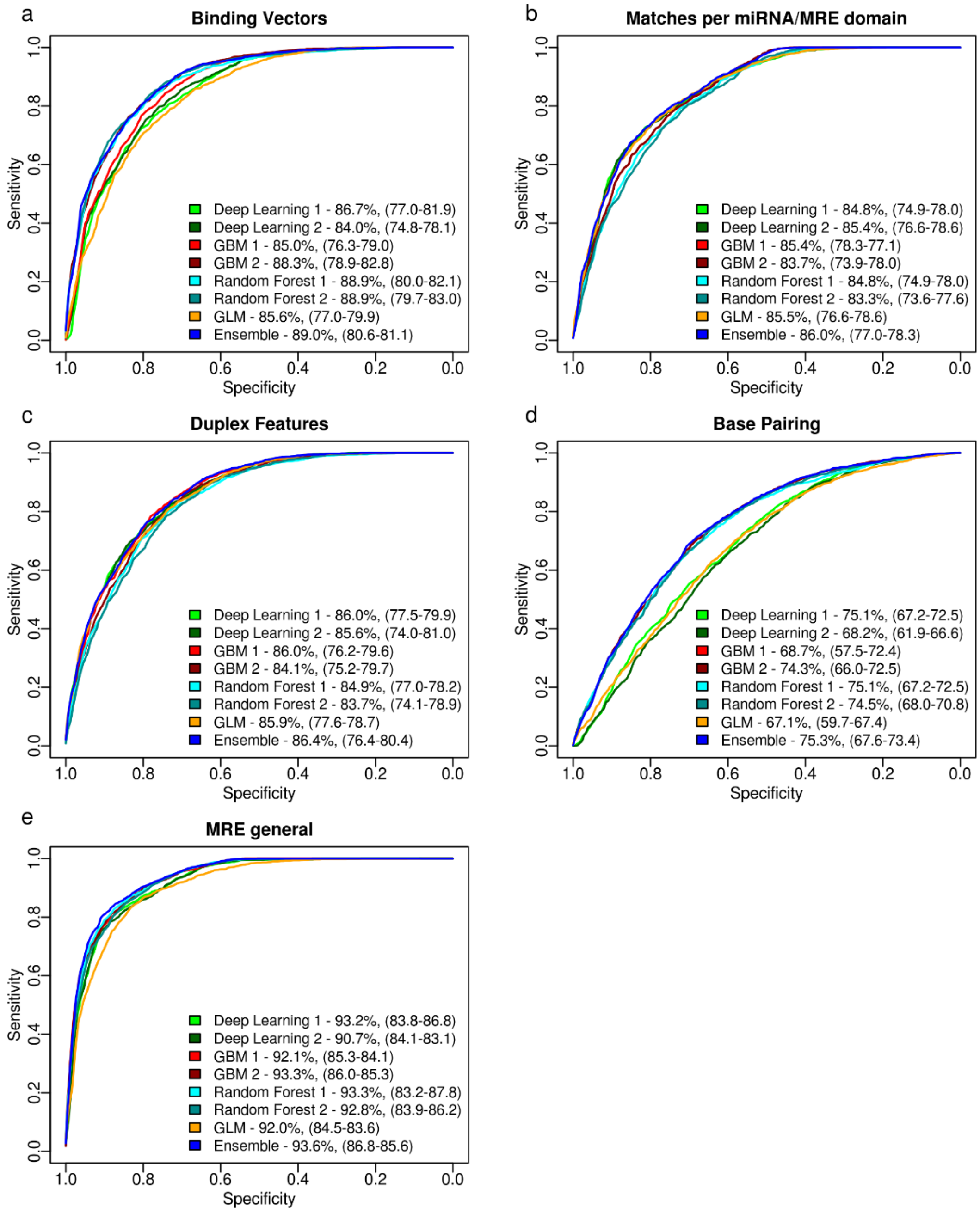

Evaluation of constitutive/internal classifiers of 5 microCLIP base models that adopt a super learning approach. Five-fold cross-validation was applied on a separate set (same as in Supplementary Fig. 10), to test the performance of the seven individual Random Forest (RF), Generalized Linear Model (GLM), Gradient Boosting Model (GBM), Deep Learning (DL) classifiers (2 RF, 2 GBM, 2 DL, 1 GLM

models) in each base node. Different colors are consistently utilized to display ROC curves of each sub-classifier incorporated in 'Binding Vectors', 'Matches per miRNA/MRE domain', 'Duplex Features', 'Base pairing' and 'MRE general' base nodes respectively. Information concerning sensitivity, specificity and AUC of each model is shown in the figure legends. The performance of ensemble deep learning models that aggregate the seven independent sub-classifiers in each base node are additionally shown.

Supplementary Figure 12

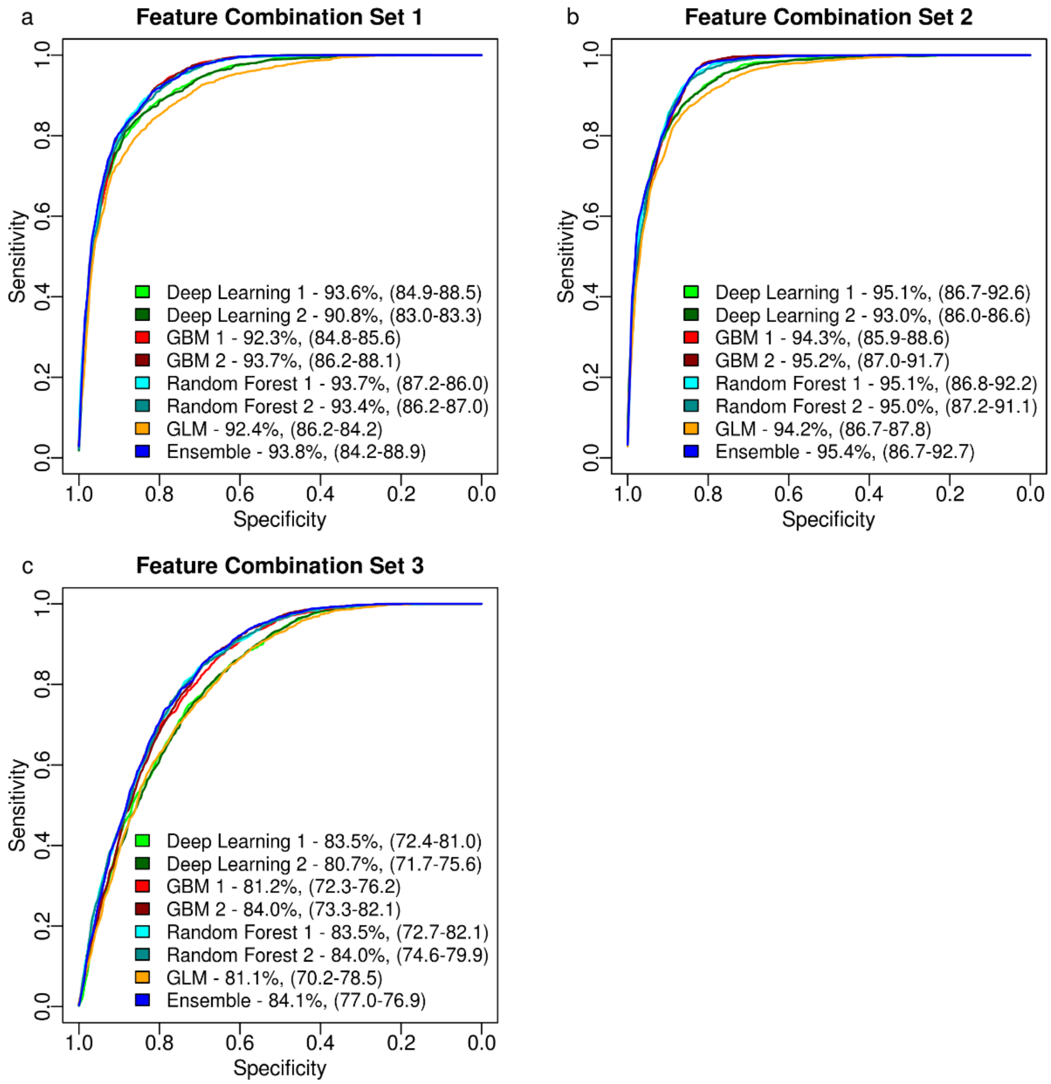

Evaluation of the accuracy of sub-classifiers included in 'Feature Combination Set 1-3' base nodes. The performance of sub-classifiers (2 RF, 2 GBM, 2 DL, 1 GLM models), along with the performance of the ensemble deep learning models that aggregate their output are displayed in distinct colors (see Supplementary Fig. 10 and 11 for more information about the ROC curves).

Supplementary Figure 13

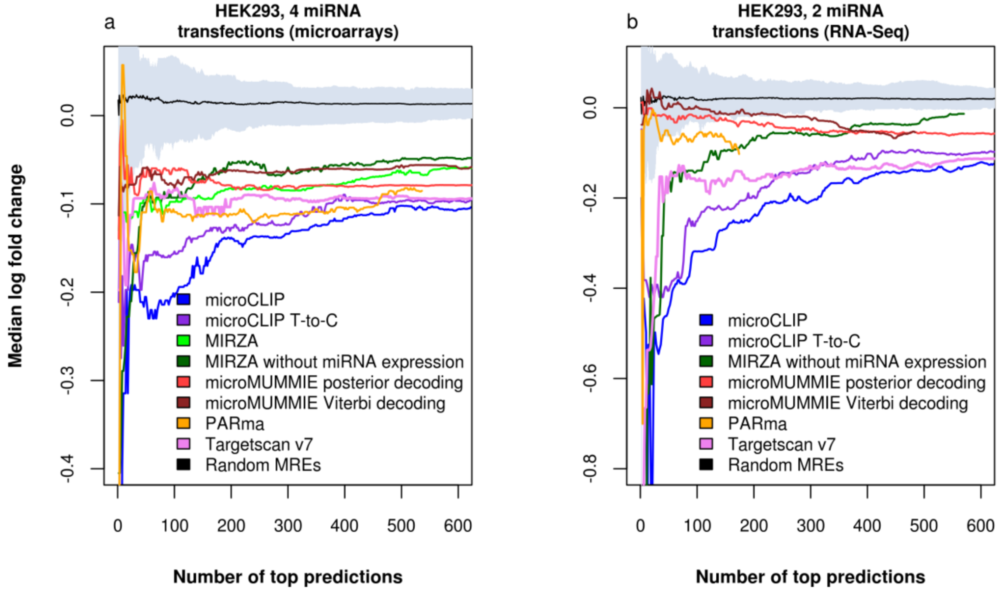

Prediction efficacy of microCLIP, microCLIP T-to-C, MIRZA, microMUMMIE, PARma and Targetscan v7. miRNA-target pairs for each *in silico* approach were obtained from the analysis of 7 AGO-PAR-CLIP HEK293 libraries and functional investigation was performed by measuring mRNA responses to miRNA perturbations. Unified sets of (a) 4 microarray and (b) 2 RNA-Seq datasets, in which miRNAs were individually transfected into HEK293 cells, were included in the evaluation process. Median fold change-values (log_2) of the top predicted targets per tested algorithm were plotted and accordingly compared by applying stepwise cutoffs on total predictions. A group comprising median fold changes of 1000 randomly selected genes (without replacement) by using 100 re-samplings is also incorporated (Random MREs). The grey shaded area represents the minimum-to-maximum log_2 fold change range of the re-samplings per number of top predictions. microCLIP outperforms microCLIP T-to-C, detecting targets with the strongest downregulation on average, from stringent to loose prediction thresholds. microCLIP T-to-C exhibits greater efficacy than the rest *in silico* approaches in (a) the 4 microarray set and (b) the 2 RNA-Seq miRNA transfection experiments (range of P values $_{\text{microarrays}}$: 0 – $2.2\text{x}10^{-7}$, P values $_{\text{RNA-Seq}}$: $5.5\text{x}10^{-265}$ – $3.6\text{x}10^{-29}$, two-tailed Wilcoxon signed-rank test, $535 < n_{\text{microarrays}} < 3{,}223$, $174 < n_{\text{RNA-Seq}} < 1{,}613$).

Supplementary Table 1

Summary of the collected AGO-PAR-CLIP experiments in human species, obtained from 9 studies. These datasets provided the source of PAR-CLIP signal (raw reads and transitions) which was integrated with experimentally validated positive/negative instances of miRNA-targeted regions.

Accession	Repository	Authors	Experiment	Species	Cell line	Samples
**GSE28859**	ncbi.nlm.nih.gov/geo	Kishore *et al.*	PAR-CLIP	human	HEK293	GSM714644, GSM714645, GSM714646, GSM714647
**SRR1045082**	ncbi.nlm.nih.gov/sra	Farazi *et al.*	PAR-CLIP	human	MCF7	SRA110557
**SRR359787**	ncbi.nlm.nih.gov/sra	Lipchina *et al.*	PAR-CLIP	human	hESC	SRA047324
**GSE59944**	ncbi.nlm.nih.gov/geo	Whisnant *et al.*	PAR-CLIP	human	C8166	GSM1462572
**GSE59944**	ncbi.nlm.nih.gov/geo	Whisnant *et al.*	PAR-CLIP	human	TZMBL	GSM1462573, GSM1462574
**GSE32109**	ncbi.nlm.nih.gov/geo	Gottwein *et al.*	PAR-CLIP	human	BC-1	GSM796037, GSM796038
**GSE32109**	ncbi.nlm.nih.gov/geo	Gottwein *et al.*	PAR-CLIP	human	BC-3	GSM796039, GSM796040
**GSE41437**	ncbi.nlm.nih.gov/geo	Skalsky *et al.*	PAR-CLIP	human	EF3D-AGO2	GSM1020021
**GSE41437**	ncbi.nlm.nih.gov/geo	Skalsky *et al.*	PAR-CLIP	human	LCL35	GSM1020022
**GSE41437**	ncbi.nlm.nih.gov/geo	Skalsky *et al.*	PAR-CLIP	human	LCL-BAC	GSM1020023
**GSE41437**	ncbi.nlm.nih.gov/geo	Skalsky *et al.*	PAR-CLIP	human	LCL-BAC-D1	GSM1020024
**GSE41437**	ncbi.nlm.nih.gov/geo	Skalsky *et al.*	PAR-CLIP	human	LCL-BAC-D3	GSM1020025
**GSE21578**	ncbi.nlm.nih.gov/geo	Hafner *et al.*	PAR-CLIP	human	HEK293	GSM545212, GSM545213, GSM545214, GSM545215
**GSE43573**	ncbi.nlm.nih.gov/geo	Memczak *et al.*	PAR-CLIP	human	HEK293	GSM1065667, GSM1065668, GSM1065669, GSM1065670
**GSE43909**	ncbi.nlm.nih.gov/geo	Erhard *et al.*	PAR-CLIP	human	BCBL-1	GSM1074233, GSM1074234

Supplementary Table 2

Overview of miRNA-target positive/negative instances as identified by different indirect/direct, low and high-throughput experiments. miRNA-targeted regions presented an overlap with AGO-bound enriched regions from at least one PAR-CLIP sequencing library. No overlap was allowed between positive and negative miRNA-gene interactions and their related MRE-instances.

**Positive Instances**	**miRNAs in interactions**	**miRNA-target pairs**
Chimeric	239	6,466
Reporter	108	258
RNA-Seq	2	1,072
Microarrays	43	2,187
Biotin pull-down	7	2,278
AGO-IP	3	351
pSILAC	5	154
RPF	2	1,899
**Negative Instances**	**miRNAs in interactions**	**miRNA-target pairs**
RNA-Seq	2	818
Microarrays	44	2,412
pSILAC	5	67
RPF	2	3,827
Background CLIP-Seq	393	24,166

Supplementary Table 3

Summary of training/test sets utilized for microCLIP deployment. The collection of positive/negative miRNA-target interactions has been derived by different indirect/direct low and high-throughput experiments, combined with AGO-bound regions from 24 PAR-CLIP libraries. Negative MRE sites were additionally extracted from an assembly of 3 background PAR-CLIP libraries expressing a commonly utilized non-RBP control (FLAG-GFP). Data set collection and MRE site extraction is described in Online Methods and Figure 2.

	**miRNAs in interactions**		**miRNA-target pairs**	
	Training	Test	Training	Test
**Positive Instances**				
Direct Techniques	244	158	4,707	2,017
miRNA perturbation experiments	47	5	7,262	679
**Negative Instances**				
Background CLIP-Seq	393	122	22,575	1,591
miRNA perturbation experiments	44	23	5,916	1,208

Supplementary Table 4

Summary of the collected microarray experiments in human species upon specific miRNA deregulation. The datasets were utilized to extract independent training and test sets of positive and negative MRE regions for microCLIP deployment.

Accession	Repository	Authors	Cell Type	miRNA	miRNA treatment	Post-Transfection Cell Harvest Time/Experimental Condition
**GSE27718**	ncbi.nlm.nih.gov/geo	Gaziel-Sovran *et al.*	113/6-4L, 131/4-5B1	hsa-miR-30d-5p	Overexpression	60h
**GSE58004**	ncbi.nlm.nih.gov/geo	Kiga *et al.*	AGS	hsa-miR-210-3p	Overexpression	36h
**GSE38956**	ncbi.nlm.nih.gov/geo	Ramachandran *et al.*	CALU3	hsa-miR-138-5p	Overexpression	48h
**GSE12400**	ncbi.nlm.nih.gov/geo	Sander *et al.*	CCL86, CRL1432	hsa-miR-26a-5p	Overexpression	72h
**GSE51053**	ncbi.nlm.nih.gov/geo	Kristensen *et al.*	DU145	hsa-miR-452-5p	Overexpression	48h
**GSE42823**	ncbi.nlm.nih.gov/geo	Nelson *et al.*	H4	hsa-miR-103a-3p	Overexpression	48h
**GSE42823**	ncbi.nlm.nih.gov/geo	Nelson *et al.*	H4	hsa-miR-107	Overexpression	48h
**GSE42823**	ncbi.nlm.nih.gov/geo	Nelson *et al.*	H4	hsa-miR-15b-3p	Overexpression	48h
**GSE42823**	ncbi.nlm.nih.gov/geo	Nelson *et al.*	H4	hsa-miR-16-5p	Overexpression	48h
**GSE42823**	ncbi.nlm.nih.gov/geo	Nelson *et al.*	H4	hsa-miR-195-5p	Overexpression	48h
**GSE42823**	ncbi.nlm.nih.gov/geo	Nelson *et al.*	H4	hsa-miR-320b	Overexpression	48h
**GSE22790**	ncbi.nlm.nih.gov/geo	Elyakim *et al.*	HEPG2	hsa-miR-191-5p	Anti-miR	NA
**GSE6207**	ncbi.nlm.nih.gov/geo	Wang *et al.*	HEPG2	hsa-miR-124-3p	Overexpression	4h, 8h, 16h, 24h, 32h, 72h , 120h
**GSE56973**	ncbi.nlm.nih.gov/geo	Hill *et al.*	HEY	hsa-miR-429	Overexpression	48h
**GSE23392**	ncbi.nlm.nih.gov/geo	Shahab *et al.*	HEY	hsa-miR-128-3p	Overexpression	48h
**GSE23392**	ncbi.nlm.nih.gov/geo	Shahab *et al.*	HEY	hsa-miR-7-5p	Overexpression	48h
**GSE41737**	ncbi.nlm.nih.gov/geo	Shirasaki *et al.*	HUH7.5	hsa-miR-27a-3p	Anti-miR, Overexpression	NA
**GSE16962**	ncbi.nlm.nih.gov/geo	Fasanaro *et al.*	HUVEC	hsa-miR-210-3p	Anti-miR, Overexpression	24h
**GSE18651**	ncbi.nlm.nih.gov/geo	Cushing *et al.*	IMR90	hsa-miR-29a-3p	Knockdown	48h
**GSE16674**	ncbi.nlm.nih.gov/geo	Navarro *et al.*	K562	hsa-miR-34a-5p	Overexpression	24h
**GSE17362**	ncbi.nlm.nih.gov/geo	Boll *et al.*	LNCAP	hsa-miR-130a-3p	Overexpression	24h
**GSE17362**	ncbi.nlm.nih.gov/geo	Boll *et al.*	LNCAP	hsa-miR-203a-3p	Overexpression	24h
**GSE17362**	ncbi.nlm.nih.gov/geo	Boll *et al.*	LNCAP	hsa-miR-205-5p	Overexpression	24h
**GSE31620**	ncbi.nlm.nih.gov/geo	Hudson *et al.*	LNCAP	hsa-miR-1	Overexpression	24h
**GSE31620**	ncbi.nlm.nih.gov/geo	Hudson *et al.*	LNCAP	hsa-miR-27b-3p	Overexpression	24h
**GSE33538**	ncbi.nlm.nih.gov/geo	Bossel Ben-Moshe *et al.*	MCF10A	hsa-miR-20a-5p	Silencing	0h, 0.5h, 1h, 2h
**GSE33538**	ncbi.nlm.nih.gov/geo	Bossel Ben-Moshe *et al.*	MCF10A	hsa-miR-671-5p	Silencing	0h, 1h, 2h
**GSE58142**	ncbi.nlm.nih.gov/geo	Frankel *et al.*	MCF7	hsa-miR-95a-3p	Overexpression	24h
**GSE31397**	ncbi.nlm.nih.gov/geo	Frankel *et al.*	MCF7	hsa-miR-101-3p	Overexpression	24h
**GSE19777**	ncbi.nlm.nih.gov/geo	Rao *et al.*	MCF7FR	hsa-miR-221-3p	Silencing	72h
**GSE58004**	ncbi.nlm.nih.gov/geo	Kiga *et al.*	MKN45	hsa-miR-210-3p	Overexpression	36h

**GSE32876**	ncbi.nlm.nih.gov/geo	Setty *et al.*	MSK543	hsa-miR-124-3p	Overexpression	24h
**GSE32876**	ncbi.nlm.nih.gov/geo	Setty *et al.*	MSK543	hsa-miR-132-3p	Overexpression	24h
**GSE57158**	ncbi.nlm.nih.gov/geo	Greenberg *et al.*	PAG C81-61	hsa-miR-20a-5p	Overexpression	3d
**GSE51053**	ncbi.nlm.nih.gov/geo	Kristensen *et al.*	PC3	hsa-miR-224-5p	Overexpression	48h
**GSE51053**	ncbi.nlm.nih.gov/geo	Kristensen *et al.*	PC3	hsa-miR-452-5p	Overexpression	48h
**GSE65892**	ncbi.nlm.nih.gov/geo	Wagenaar *et al.*	SKHEP1	hsa-miR-21-5p	Anti-miR	16h
**GSE19693**	ncbi.nlm.nih.gov/geo	Chen *et al.*	U87, HS683	hsa-miR-20a-5p	Overexpression	NA
**GSE34846**	ncbi.nlm.nih.gov/geo	Cao *et al.*	HTERT-RPE1	hsa-miR-129-2-3p	Overexpression	72h
**GSE37427**	ncbi.nlm.nih.gov/geo	Zhu *et al.*	FLS	hsa-miR-23b-3p	Overexpression	NA
**GSE22143**	ncbi.nlm.nih.gov/geo	Marcet *et al.*	HAEC	hsa-miR-34a-5p	Overexpression	48h
**GSE22143**	ncbi.nlm.nih.gov/geo	Marcet *et al.*	HAEC	hsa-miR-34c-5p	Overexpression	48h
**GSE22143**	ncbi.nlm.nih.gov/geo	Marcet *et al.*	HAEC	hsa-miR-449b-5p	Overexpression	48h
**GSE22143**	ncbi.nlm.nih.gov/geo	Marcet *et al.*	HAEC	hsa-miR-449a	Overexpression	48h
**GSE68424**	ncbi.nlm.nih.gov/geo	Teplyuk *et al.*	GBM4, GBM6	hsa-miR-10b-5p	Inhibition	24h
**GSE35621**	ncbi.nlm.nih.gov/geo	Hu *et al.*	HEK293T, HSF2	hsa-miR-941	Overexpression	24h
**GSE37596**	ncbi.nlm.nih.gov/geo	Hwang *et al.*	HT29	hsa-miR-146a-5p	Overexpression	2w after lentiviral infection
**GSE40058**	ncbi.nlm.nih.gov/geo	Luo *et al.*	MDA-MB-231	hsa-miR-200c-3p	Overexpression	NA
**GSE40058**	ncbi.nlm.nih.gov/geo	Luo *et al.*	MDA-MB-231	hsa-miR-205-5p	Overexpression	NA
**GSE7754**	ncbi.nlm.nih.gov/geo	Chang *et al.*	HCT116	hsa-miR-34a-5p	Overexpression	2w after retroviral infection
**GSE51875**	ncbi.nlm.nih.gov/geo	Lee *et al.*	HCT116	hsa-miR-147a	Overexpression	3d
**GSE50697**	ncbi.nlm.nih.gov/geo	Taube *et al.*	SUM159	hsa-miR-203a-3p	Overexpression	NA
**GSE35208**	ncbi.nlm.nih.gov/geo	Lin *et al.*	U87-2M1	hsa-miR-10b-5p	Inhibition	NA
**GSE14507**	ncbi.nlm.nih.gov/geo	Webster *et al.*	A549	hsa-miR-7-5p	Overexpression	24h
**GSE21132**	ncbi.nlm.nih.gov/geo	Li *et al.*	Jurkat	hsa-miR-146a-5p	Overexpression, Knockdown	48h
**GSE24824**	ncbi.nlm.nih.gov/geo	Huynh *et al.*	Melanoma-metastatic Liver Cells	hsa-miR-182-5p	Anti-miR	administered twice per week over 4 weeks
**GSE56268**	ncbi.nlm.nih.gov/geo	Schneider *et al.*	P3HR1	hsa-miR-28-5p	Overexpression	12h, 24h

Supplementary Table 5

Summary of the collected RNA Sequencing experiments in human species upon miRNA overexpression. The datasets were utilized to extract independent training and test sets of positive and negative MRE regions for microCLIP deployment.

Accession	Repository	Authors	Cell Type	miRNA	miRNA treatment	Post-Transfection Cell Harvest Time/Experimental

						Condition
**GSE52531**	ncbi.nlm.nih.gov/geo	Nam *et al.*	HEK293	hsa-miR-155-5p	Overexpression	24h
**GSE60426**	ncbi.nlm.nih.gov/geo	Eichhorn *et al.*	HeLa	hsa-miR-155-5p	Overexpression	32h
**GSE60426**	ncbi.nlm.nih.gov/geo	Eichhorn *et al.*	U2OS (total)	hsa-miR-155-5p	Overexpression	32h/poly(A)-selected total RNA
**GSE60426**	ncbi.nlm.nih.gov/geo	Eichhorn *et al.*	U2OS (cyto)	hsa-miR-155-5p	Overexpression	32h/poly(A)-selected cytoplasmic RNA
**GSE60426**	ncbi.nlm.nih.gov/geo	Eichhorn *et al.*	U2OS (ribo)	hsa-miR-155-5p	Overexpression	tRNA and rRNA depleted RNA
**GSE37918**	ncbi.nlm.nih.gov/geo	Pellegrino *et al.*	MDA-MB-231	hsa-miR-23b-3p	Overexpression	NA

Supplementary Table 6

Summary of microarray and RNA sequencing experiments in human species upon specific miRNA deregulation, utilized in benchmarking evaluations of microCLIP model (Fig. 5-7, Supplementary Fig. 13).

Accession	Repository	Authors	Cell Type	miRNA	miRNA treatment	Post-Transfection Cell Harvest Time/Experimental Condition
**GSE46039**	ncbi.nlm.nih.gov/geo	Helwak *et al.*	HEK293	hsa-miR-92a-3p	Knockdown	48h
**GSE21577**	ncbi.nlm.nih.gov/geo	Hafner *et al.*	HEK293	hsa-miR-20a-5p	Knockdown	simultaneous miRNA knockdown using inhibitor cocktail
**GSE21901**	ncbi.nlm.nih.gov/geo	Hollander *et al.*	HEK293	hsa-miR-212-3p	Overexpression	NA
**GSE14537**	ncbi.nlm.nih.gov/geo	Hausser *et al.*	HEK293	hsa-miR-124-3p	Overexpression	15h
**GSE14537**	ncbi.nlm.nih.gov/geo	Hausser *et al.*	HEK293	hsa-miR-7-5p	Overexpression	15h
**GSE35621**	ncbi.nlm.nih.gov/geo	Hu *et al.*	HEK293	hsa-miR-941	Overexpression	24h
**NA**	psilac.mdc-berlin.de	Selbach *et al.*	HeLa	hsa-let-7b-5p	Overexpression	32h
**NA**	psilac.mdc-berlin.de	Selbach *et al.*	HeLa	hsa-miR-1	Overexpression	32h
**NA**	psilac.mdc-berlin.de	Selbach *et al.*	HeLa	hsa-miR-155-5p	Overexpression	32h
**NA**	psilac.mdc-berlin.de	Selbach *et al.*	HeLa	hsa-miR-16-5p	Overexpression	32h
**NA**	psilac.mdc-berlin.de	Selbach *et al.*	HeLa	hsa-miR-30a-5p	Overexpression	32h
**GSE8501**	ncbi.nlm.nih.gov/geo	Grimson *et al.*	HeLa	hsa-miR-7-5p	Overexpression	24h
**GSE52531**	ncbi.nlm.nih.gov/geo	Nam *et al.*	HEK293	hsa-miR-124-3p	Overexpression	24h
**GSE68987**	ncbi.nlm.nih.gov/geo	Zhang *et al.*	HeLa	hsa-miR-603	Overexpression	24h
**GSE52531**	ncbi.nlm.nih.gov/geo	Nam *et al.*	HeLa	hsa-miR-155-5p	Overexpression	24h
**GSE52531**	ncbi.nlm.nih.gov/geo	Nam *et al.*	HeLa	hsa-miR-124-3p	Overexpression	24h
**GSE60426**	ncbi.nlm.nih.gov/geo	Eichhorn *et al.*	HEK293T	hsa-miR-1	Overexpression	24h
**GSE37918**	ncbi.nlm.nih.gov/geo	Pellegrino *et al.*	MCF7	hsa-miR-23b-3p	Overexpression	NA

Supplementary Table 7

Summary of the collected ribosome profiling sequencing experiments (RPF-Seq) in human species after overexpression of a specific miRNA. The datasets were utilized to extract independent training and test sets of positive and negative MRE regions for microCLIP deployment.

Accession	Repository	Authors	Cell Type	miRNA	miRNA treatment	Post-Transfection Cell Harvest Time/Experimental Condition
**GSE60426**	ncbi.nlm.nih.gov/geo	Eichhorn *et al.*	HEK293T	hsa-miR-1-3p	Overexpression	24h
**GSE60426**	ncbi.nlm.nih.gov/geo	Eichhorn *et al.*	HeLa	hsa-miR-155-5p	Overexpression	32h
**GSE60426**	ncbi.nlm.nih.gov/geo	Eichhorn *et al.*	U2OS	hsa-miR-1-3p	Overexpression	32h
**GSE60426**	ncbi.nlm.nih.gov/geo	Eichhorn *et al.*	U2OS	hsa-miR-155-5p	Overexpression	32h
**GSE60426**	ncbi.nlm.nih.gov/geo	Eichhorn *et al.*	HeLa	hsa-miR-1-3p	Overexpression	32h

Supplementary Table 8

Summary of the collected pSILAC experiments upon miRNA deregulation (miRNA overexpression or knockdown). The datasets were utilized to extract independent training and test sets of positive and negative MRE regions for microCLIP deployment.

Repository	Authors	Cell Type	miRNA	miRNA treatment	Post-Transfection Cell Harvest Time/Experimental Condition
**psilac.mdc-berlin.de**	Selbach *et al.*	HeLa	hsa-let-7b-5p	Overexpression, Knockdown	8h post-transfection and 24h pSILAC labelling
**psilac.mdc-berlin.de**	Selbach *et al.*	HeLa	hsa-miR-1-3p	Overexpression	8h post-transfection and 24h pSILAC labelling
**psilac.mdc-berlin.de**	Selbach *et al.*	HeLa	hsa-miR-16-5p	Overexpression	8h post-transfection and 24h pSILAC labelling
**psilac.mdc-berlin.de**	Selbach *et al.*	HeLa	hsa-miR-30a-5p	Overexpression	8h post-transfection and 24h pSILAC labelling
**psilac.mdc-berlin.de**	Selbach *et al.*	HeLa	hsa-miR-155-5p	Overexpression	8h post-transfection and 24h pSILAC labelling

Supplementary Table 9

Summary of the collected Biotin pull-down and AGO-IP experiments upon miRNA perturbation. The datasets were utilized to extract independent training and test sets of positive MRE regions for microCLIP deployment.

Accession	Repository	Authors	Experiment	Cell Type	miRNA	miRNA treatment	Post-Transfection Cell Harvest Time
**GSE40408**	ncbi.nlm.nih.gov/geo	Martin et al.	Biotin pull-down	HEK293T	hsa-miR-23b-3p	Overexpression	24h
**GSE40408**	ncbi.nlm.nih.gov/geo	Martin et al.	Biotin pull-down	HEK293T	hsa-miR-27a-3p	Overexpression	24h
**GSE40408**	ncbi.nlm.nih.gov/geo	Martin et al.	Biotin pull-	HEK293T	hsa-miR-17-5p	Overexpression	24h

			down				
**GSE29101**	ncbi.nlm.nih.gov/geo	Cloonan et al.	Biotin pull-down	HEK293T	hsa-miR-10a-5p	Overexpression	24h
**GSE40411**	ncbi.nlm.nih.gov/geo	Krishnan et al.	Biotin pull-down	MCF7	hsa-miR-139-5p	Overexpression	24h
**GSE38593**	ncbi.nlm.nih.gov/geo	Krishnan et al.	Biotin pull-down	HEK293T	hsa-miR-182-5p	Overexpression	24h
**GSE11082**	ncbi.nlm.nih.gov/geo	Hendrickson et al.	AGO-IP	HEK293T	hsa-miR-1	Overexpression	48h
**GSE11082**	ncbi.nlm.nih.gov/geo	Hendrickson et al.	AGO-IP	HEK293T	hsa-miR-124-3p	Overexpression	48h
**GSE39227**	ncbi.nlm.nih.gov/geo	Hu et al.	AGO-IP	HEK293T	hsa-miR-941	Overexpression	NA
**NA**	doi:10.1371/journal.pgen.1002363.s006	Lal et al.	Biotin pull-down	K562	hsa-miR-34a-5p	Overexpression	24h
**NA**	doi:10.1371/journal.pgen.1002363.s006	Lal et al.	Biotin pull-down	HTC116	hsa-miR-34a-5p	Overexpression	24h

Supplementary Table 10

Description of small RNA-Seq datasets of similar cell types to PAR-CLIP libraries, analyzed to infer expressed miRNAs. The table displays the source of small RNA-Seq libraries along with its ID, cell type, condition and description.

Accession	Repository	Cell Type/Tissue	Description
**GSM897079_Rep1**	ncbi.nlm.nih.gov/geo	HeLaS3	Cervical Carcinoma
**GSM897079_Rep2**	ncbi.nlm.nih.gov/geo	HeLaS3	Cervical Carcinoma
**GSM897073_Rep1**	ncbi.nlm.nih.gov/geo	H1hESC	Embryonic Stem Cells
**GSM897073_Rep2**	ncbi.nlm.nih.gov/geo	H1hESC	Embryonic Stem Cells
**GSM973690_Rep3**	ncbi.nlm.nih.gov/geo	MCF7	Adenocarcinoma
**GSM973690_Rep4**	ncbi.nlm.nih.gov/geo	MCF7	Adenocarcinoma
**GSM897081_Rep1**	ncbi.nlm.nih.gov/geo	MCF7	Adenocarcinoma
**GSM897081_Rep2**	ncbi.nlm.nih.gov/geo	MCF7	Adenocarcinoma
**SRR2084358**	ncbi.nlm.nih.gov/sra	MCF7	Adenocarcinoma
**GSM1020026**	ncbi.nlm.nih.gov/geo	EF3D-AGO2	Adenocarcinoma
**GSM1020028**	ncbi.nlm.nih.gov/geo	LCL-BAC	Adenocarcinoma
**GSM1020029**	ncbi.nlm.nih.gov/geo	LCL-BAC-D1	Adenocarcinoma
**GSM1020030**	ncbi.nlm.nih.gov/geo	LCL-BAC-D3	Adenocarcinoma

Supplementary Table 11

Description of RNA-Seq datasets of similar cell types to PAR-CLIP libraries, analyzed to infer expressed transcripts. The table displays the source of RNA-Seq datasets along with its ID, cell type, condition and description.

Accession	Repository	Cell Type/Tissue	Condition	Description
**SRR837795**	ncbi.nlm.nih.gov/sra	LCL-BAC-D1	miR-BHRF1-1 mutant virus	LCL infected with an EBV B95-8 BACmid

**SRR837796**	ncbi.nlm.nih.gov/sra	LCL-BAC-D1	miR-BHRF1-1 mutant virus	LCL infected with an EBV B95-8 BACmid
**SRR837797**	ncbi.nlm.nih.gov/sra	LCL-BAC-D2	miR-BHRF1-2 mutant virus	LCL infected with an EBV B95-8 BACmid
**SRR837798**	ncbi.nlm.nih.gov/sra	LCL-BAC-D3	miR-BHRF1-3 mutant virus	LCL infected with an EBV B95-8 BACmid
**SRR837794**	ncbi.nlm.nih.gov/sra	LCL-BAC	NA	LCL infected with an EBV B95-8 BACmid
**ENCFF002DKY & ENCFF002DKX**	encodeproject.org	MCF7	NA	Adenocarcinoma
**ENCFF000FOV & ENCFF000FOM**	encodeproject.org	HeLaS3	NA	Cervical Carcinoma
**wgEncodeCshlLongRnaSeqH1hescCellPapFastqRep1**	hgdownload.cse.ucsc.edu	H1hESC	NA	Embryonic Stem Cells
**GSM1370364**	ncbi.nlm.nih.gov/geo	HEK293	NA	Embryonic Kidney Cells

Supplementary Note 1

Context-dependency and cell-type specificity are indeed important factors of microRNA function and are central to CLIP-derived miRNA target identification[1]. We curated and incorporated data from numerous experiments and cell-types to avoid confining our implementation to a specific context.

In order to examine the context-specificity of non-T-to-C sites and to investigate whether this is another point of concordance with T-to-C sites, we followed the seminal experiment performed in Erhard *et al.*[2]. We analyzed three B-cell lines latently infected with Kaposi's sarcoma-associated herpes virus (KSHV), namely BCBL1 (Erhard *et al.*[2]), BC1 and BC3 (Gottwein *et al.*[3]). We performed a first exploratory analysis of KSHV-encoded miRNA context-dependency. KSHV miRNAs have been shown to have the same preferences as cellular miRNAs (Erhard *et al.*[1]). In brief, we isolated PAR-CLIP enriched regions in protein coding transcripts that are targeted by KSHV microRNAs and present >= 5 normalized reads in at least one library. For appropriate comparison of the libraries, read counts were normalized based on the geometric mean across all samples as described by Anders *et al.*[4]. The analysis was realized on 10,474 T-to-C and 2,323 non-T-to-C regions that are mutually exclusive. Heatmap representation of (non-)T-to-C sites shows high correlation and reproducibility of targeted regions between replicated PAR-CLIP libraries, while comparison across B-cell lines uncovers a smaller overlap (Supplementary Fig. 1a). We additionally visualized in the same viral miRNA-targeted regions the normalized read coverage of 4 lymphoblastoid EBV infected cell lines (LCL-BAC, LCL-BAC-D1, LCL-BAC-D3 and LCL35) from the study of Skalsky *et al.*[5]. The latter PAR-CLIP libraries, as we initially hypothesized, present no correlation and significantly less enrichment on most of the KSHV miRNA context-dependent (non-)T-to-C sites (Supplementary Fig. 1b).

Supplementary Note 2

The enrichment of KEGG pathways was interrogated with miRNA targets from a PAR-CLIP data set in MCF7 cells. Despite the gene set site increase ($n_{\text{T-to-C targets}}$ = 396, $n_{\text{(non-)T-to-C targets}}$ = 491), assessing the impact of non-T-to-C clusters in defining miRNA regulation of pathways yielded similar or lower enrichment P values (Supplementary Fig. 4). As discussed in section 'Functional enrichment analysis reveals importance of non-T-to-C supported targets', complementing the gene set with targets lacking sufficient T-to-C substitutions reveals additional pathway nodes to be under miRNA regulation. Genes found by (non-)T-to-C clusters to be targeted in Hippo, TGF-beta, FoxO signaling pathways and Adherens Junction are commented below in the context of breast cancer. However, further experiments should be conducted to validate and accurately measure this information gain with respect to pathways.

In Hippo signaling pathway, non-T-to-C peaks uncovered miRNA-induced regulation of YWHAE (14-3-3ε), ACTB and ACTG1 (F-actin), MPP5 (Pals1) and DLG3 (Dlg), responsible for cytoplasmic retention/proteolysis of YAP/TAZ[6,7] and of positive regulator PPP2R2C (PP2A)[6] (Supplementary Fig. 5). YAP/TAZ complex translocates into the nucleus under low cell density conditions to promote cell growth and proliferation. miRNA targeting of nodes 14-3-3 and PP2A was shown by T-to-C clusters for family members YWHAH, YWHAZ and PPP1CC, PPP2CA,

PPP2CB, PPP2R1B respectively. In the Frizzled receptor class, T-to-C peaks indicated negative regulator of canonical Wnt/beta-catenin signaling FZD6[8], and non-T-to-C peaks additionally denoted positive regulator FZD3[8] as miRNA targets. Additionally, BIRC5 (Survivin), a terminal Hippo pathway node exerting pro-proliferative and anti-apoptotic roles, was also highlighted as a target from the non-T-to-C peaks.

Enrichment analysis with T-to-C-containing genes revealed targeting of main effector molecules TGFBR1 and SMADs (SMAD2, SMAD4, SMAD5 and SMAD7), as well as of ubiquitin-protein ligase SMURF2, in the TGF-beta signaling pathway. The non-T-to-C clusters added TGIF2, TFDP-1, SMURF1 and EP300 as miRNA targets (Supplementary Fig. 5). TGIF2 and transcription factor DP-1 are repressors of TGF-beta-dependent transcription that regulate cell-cycle genes[9,10]. SMURF1 induces cell motility and metastasis in breast cancer cells[11] while EP300 co-binds with SP-1 and Smad2/3 to induce TGF-beta-dependent transcription[12].

In FoxO signaling pathway, apart from BCL2L11 (Bim), other pathway-related genes identified from non-T-to-C peaks were EP300 (CBP), responsible for FOXO-1 transcriptional activation[13] and IKBKB, a cytoplasmic FOXO3a repressor promoting cell growth and tumorigenesis[14]. IKBKB forms IKKα/β active kinase complex with CHUK[14], shown from T-to-C peaks to be targeted. Enrichment analysis of the T-to-C group additionally highlighted miRNA interactions with EGFR and Insulin receptor signaling genes related to cytoplasmic FOXO repression (Supplementary Fig. 6).

A pathway ranking higher in the combined gene set compared to the T-to-C set was `Adherens junction' (hsa04520, $P_{\text{T-to-C}} = 2.59\times10^{-3}$, $\text{rank}_{\text{T-to-C}}$: 48th, $P_{\text{(non-)T-to-C}} = 5.36\times10^{-5}$, $\text{rank}_{\text{(non-)T-to-C}}$: 20th, one-sided Fisher's exact test, Benjamini-Hochberg adjustment, $n_{\text{T-to-C}} = 13$, $n_{\text{(non-)T-to-C}} = 18$). Targeted genes included MAPK and TGF-beta signaling pathway members promoting cell growth and differentiation under `Weak adhesion' Cadherin signaling (MAPK1, MAP3K7 and TGFBR1, SMAD2, SMAD4, EP300 respectively). Cadherin signaling is associated with regulation of migratory capacity and invasiveness in breast cancer[15]. miRNA targeting of weakening adhesion mediators IQGAP[16], CSNK2A2 (CKII), known to suppress cell death in breast cancer cells[17], EP300 (CBP), as well as ACTB and ACTG1 was exclusively dictated by non-T-to-C peaks (Supplementary Fig. 6).

Supplementary References

1 Erhard, F. *et al.* Widespread context dependency of microRNA-mediated regulation. *Genome research* **24**, 906-919, doi:10.1101/gr.166702.113 (2014).

2 Erhard, F., Dolken, L., Jaskiewicz, L. & Zimmer, R. PARma: identification of microRNA target sites in AGO-PAR-CLIP data. *Genome Biol* **14**, R79, doi:10.1186/gb-2013-14-7-r79 (2013).

3 Gottwein, E. *et al.* Viral microRNA targetome of KSHV-infected primary effusion lymphoma cell lines. *Cell host & microbe* **10**, 515-526, doi:10.1016/j.chom.2011.09.012 (2011).

4 Anders, S. & Huber, W. Differential expression analysis for sequence count data. *Genome Biol* **11**, R106, doi:10.1186/gb-2010-11-10-r106 (2010).

5 Skalsky, R. L. *et al.* The viral and cellular microRNA targetome in lymphoblastoid cell lines. *PLoS Pathog* **8**, e1002484, doi:10.1371/journal.ppat.1002484 (2012).

6 Johnson, R. & Halder, G. The two faces of Hippo: targeting the Hippo pathway for regenerative medicine and cancer treatment. *Nat Rev Drug Discov* **13**, 63-79, doi:10.1038/nrd4161 (2014).

7 Katoh, M. Function and cancer genomics of FAT family genes (review). *Int J Oncol* **41**, 1913-1918, doi:10.3892/ijo.2012.1669 (2012).

8 Ueno, K., Hirata, H., Hinoda, Y. & Dahiya, R. Frizzled homolog proteins, microRNAs and Wnt signaling in cancer. *Int J Cancer* **132**, 1731-1740, doi:10.1002/ijc.27746 (2013).

9 Chen, C. R., Kang, Y., Siegel, P. M. & Massague, J. E2F4/5 and p107 as Smad cofactors linking the TGFbeta receptor to c-myc repression. *Cell* **110**, 19-32 (2002).

10 Zhang, M. Z. *et al.* TGIF governs a feed-forward network that empowers Wnt signaling to drive mammary tumorigenesis. *Cancer Cell* **27**, 547-560, doi:10.1016/j.ccell.2015.03.002 (2015).

11 Yu, L. *et al.* SND1 Acts Downstream of TGFbeta1 and Upstream of Smurf1 to Promote Breast Cancer Metastasis. *Cancer Res* **75**, 1275-1286, doi:10.1158/0008-5472.CAN-14-2387 (2015).

12 Yuan, H. *et al.* Involvement of p300/CBP and epigenetic histone acetylation in TGF-beta1-mediated gene transcription in mesangial cells. *Am J Physiol Renal Physiol* **304**, F601-613, doi:10.1152/ajprenal.00523.2012 (2013).

13 Pramanik, K. C., Fofaria, N. M., Gupta, P. & Srivastava, S. K. CBP-mediated FOXO-1 acetylation inhibits pancreatic tumor growth by targeting SirT. *Mol Cancer Ther* **13**, 687-698, doi:10.1158/1535-7163.MCT-13-0863 (2014).

14 Hu, M. C. *et al.* IkappaB kinase promotes tumorigenesis through inhibition of forkhead FOXO3a. *Cell* **117**, 225-237 (2004).

15 Ganguly, K. K., Pal, S., Moulik, S. & Chatterjee, A. Integrins and metastasis. *Cell Adh Migr* **7**, 251-261, doi:10.4161/cam.23840 (2013).

16 Johnson, M., Sharma, M. & Henderson, B. R. IQGAP1 regulation and roles in cancer. *Cell Signal* **21**, 1471-1478, doi:10.1016/j.cellsig.2009.02.023 (2009).

17 Kren, B. T. *et al.* Preclinical evaluation of cyclin dependent kinase 11 and casein kinase 2 survival kinases as RNA interference targets for triple negative breast cancer therapy. *Breast Cancer Res* **17**, 19, doi:10.1186/s13058-015-0524-0 (2015).
