# Supplementary material for: microCLIP super learning framework uncovers functional transcriptome-wide miRNA interactions
Source: Nat Commun. 2018 Sep 6;9:3601. doi: 10.1038/s41467-018-06046-y (PMC6127135; doi:10.1038/s41467-018-06046-y)
Supplement: Supplementary file 2 — Description of Additional Supplementary Files [file 41467_2018_6046_MOESM2_ESM.pdf]

Description of Additional Supplementary Files

File Name: Supplementary Data 1
Description: Features incorporated in microCLIP algorithm

File Name: Supplementary Data 2
Description: Expression fold changes (log_2-transformed) in microRNA perturbation experiments used in algorithm evaluations

File Name: Supplementary Data 3
Description: Experimentally verified microRNA binding sites used in algorithm evaluations

File Name: Supplementary Data 4
Description: miRNA binding sites predicted from AGO-CLIP-guided models, utilized in the evaluation of Supplementary Figure 8
